# The economics of organellar gene loss and endosymbiotic gene transfer

**DOI:** 10.1186/s13059-021-02567-w

**Published:** 2021-12-20

**Authors:** Steven Kelly

**Affiliations:** Department of Plant Sciences, University of Oxford, South Parks Road, Oxford, OX1 3RB UK

**Keywords:** Endosymbiosis, Gene loss endosymbiotic gene transfer, Mitochondrion, Chloroplast, Organellar genome

## Abstract

**Background:**

The endosymbiosis of the bacterial progenitors of the mitochondrion and the chloroplast are landmark events in the evolution of life on Earth. While both organelles have retained substantial proteomic and biochemical complexity, this complexity is not reflected in the content of their genomes. Instead, the organellar genomes encode fewer than 5% of the genes found in living relatives of their ancestors. While many of the 95% of missing organellar genes have been discarded, others have been transferred to the host nuclear genome through a process known as endosymbiotic gene transfer.

**Results:**

Here, we demonstrate that the difference in the per-cell copy number of the organellar and nuclear genomes presents an energetic incentive to the cell to either delete organellar genes or transfer them to the nuclear genome. We show that, for the majority of transferred organellar genes, the energy saved by nuclear transfer exceeds the costs incurred from importing the encoded protein into the organelle where it can provide its function. Finally, we show that the net energy saved by endosymbiotic gene transfer can constitute an appreciable proportion of total cellular energy budgets and is therefore sufficient to impart a selectable advantage to the cell.

**Conclusion:**

Thus, reduced cellular cost and improved energy efficiency likely played a role in the reductive evolution of mitochondrial and chloroplast genomes and the transfer of organellar genes to the nuclear genome.

**Supplementary Information:**

The online version contains supplementary material available at 10.1186/s13059-021-02567-w.

## Background

Endosymbiosis has underpinned two of the most important innovations in the history of life on Earth [1, 2]. The endosymbiosis of the alphaproteobacterium that became the mitochondrion led to the emergence and radiation of the eukaryotes [3–5], and the endosymbiosis of the cyanobacterium that became the chloroplast first enabled oxygenic photosynthesis in eukaryotes [6, 7]. The function and evolution of both organelles are inextricably linked with energy metabolism and the evolution of the eukaryotic cell [4, 8–14], and together they have given rise to the multicellular organisms that constitute the largest fraction of the biomass of the biosphere [15].

Following the endosymbioses of the bacterial progenitors of the mitochondrion and the chloroplast, there was a dramatic reduction in the gene content of the endosymbiont genomes such that they harbor fewer than 5% of the genes found in their free-living bacterial relatives [16–18]. While many of the original endosymbiont genes have been lost [19–22], others have been transferred to the host nuclear genome and their products imported back into the organelle where they function—a process known as endosymbiotic gene transfer [23–27]. For example, the mitochondria of humans [28] and chloroplasts of plants [29] each contain more than 1000 proteins, yet their genomes encode fewer than 100 genes. Thus, the reduced gene content of organelles is not representative of their molecular, proteomic, or biochemical complexity.

The process of gene loss and endosymbiotic gene transfer is not unique to the evolution of chloroplasts and mitochondria but has also been observed concomitant with the endosymbioses of bacteria in insects [19, 30] and the endosymbiosis of the cyanobacterium that became the chromatophore in *Paulinella* [31–35]. In addition, it has been suggested that lateral gene transfers from diverse bacteria into the host nuclear genome may have contributed to the process of organellar genome reduction in a manner that functionally recapitulates endosymbiotic gene transfer, i.e., the endosymbiont gene becomes redundant when an orthologous or functionally equivalent gene from another species is transferred to the nuclear genome [36]. Similarly, the presence of a redundant copy of a gene in the nucleus that is only slightly expressed and minimally targeted provides an opportunity for recovery in case of gene loss from the organelle. Thus, endosymbiont genome reduction in the presence of functional compensation (by lateral and/or endosymbiotic gene transfer or pre-existing nuclear genes) is a recurring theme in the evolution of organellar and endosymbiont genomes.

Given the importance of endosymbiotic gene transfer (and functionally equivalent lateral complementation) to the evolution of eukaryotic genomes, several hypotheses have been proposed to explain why it occurs [37–41]. For example, it has been proposed that lateral and endosymbiotic gene transfer protects endosymbiont genes (and the biological functions they provide) from mutational hazard [20, 21, 41, 42] and that it enables endosymbiont genes that are otherwise trapped in a haploid genome to recombine and thus escape from Muller’s ratchet [20, 21, 39, 43–45]. It has also been proposed that endosymbiotic gene transfer is an inevitable consequence of a constant stream of endosymbiont genes entering the nucleus [46–50], and that transfer to the nuclear genome allows the host cell to gain better control over the replication and function of the organelle [38] allowing better cellular network integration [33, 51]. However, mutation rates of organellar genes are often not higher than nuclear genes [20–22, 52–56], and therefore, effective mechanisms for protection against DNA damage in organelles must exist. Similarly, although there is evidence for the action of Muller’s ratchet in mitochondria [44, 45], chloroplasts appear largely to escape this effect [52, 57] likely due to gene conversion [58], and thus, it does not fully explain why endosymbiotic gene transfer occurred in both lineages. Finally, the nature of the regulatory advantage for having genes reside in the nuclear genome is difficult to quantify, as bacterial gene expression regulation is no less effective than in eukaryotes, and many eukaryotes utilize polycistronic regulation of gene expression [59–62]. Thus, it is unclear whether endosymbiotic gene transfer functions simply as rescue from processes that would otherwise lead to gene loss, or whether there may also be an advantage to the cell for transferring an endosymbiont gene to the nuclear genome.

Given the constant stream of genetic transfer to the nucleus, and the proposed reasons why these transfers may be advantageous, the question arises as to why organelles have retained any genetic material. To answer this question, several hypotheses have been put forward that suggest that there must be a selectable advantage for the retention of genes in organellar genomes. Foremost among these hypotheses is that the location of genes in organelles enables regulation of their expression by the redox state of the organelle [63–65]. In addition, analyses of thousands of organellar genomes led to the suggestion that other gene-intrinsic factors such as GC content or hydrophobicity of the gene product may also play a factor in providing an advantage for gene retention in organellar genomes [66, 67]. These collectively point to a role for natural selection in the retention of organellar genes in organellar genomes.

We hypothesized that an advantage for endosymbiotic gene transfer or retention of a gene in an organellar genome may arise from the difference in the cost to the cell of encoding a gene in the organellar and nuclear genome. This is because each eukaryotic cell typically contains multiple organelles and each organelle typically harbors multiple copies of the organellar genome [68, 69]. The number of organelles in a cell reflects the biochemical requirement of that cell for those organelles, and the high genome copy number per organelle has been proposed to provide protection against DNA damage [70] and to enable the organelle to achieve high protein abundance for genes encoded in the organellar genome [69]. Thus, while a diploid eukaryotic cell contains two copies of the nuclear genome, the same cell can contain hundreds to hundreds of thousands of copies of its organellar genomes [68, 69]. For example, endosymbiotic transfer of a 1000-bp gene from the mitochondrion to the nuclear genome in humans, yeast, or *Arabidopsis* would save 5,000,000 bp, 200,000 bp, or 100,000 bp of DNA per cell, respectively, and an analogous transfer from the chloroplast genome to the nuclear genome in *Arabidopsis* would save 1,500,000 bp of DNA per cell (see the “Methods” section for sources of genome copy numbers). As DNA costs energy and cellular resources to biosynthesize [71], we hypothesized that if the energy saved by transferring a gene from the organellar genome to the nuclear genome offset the cost of importing the encoded gene products (proteins) back into the organelle then this would provide a direct energetic advantage to the host cell for endosymbiotic gene transfer. Similarly, if a functionally equivalent gene from another species was laterally acquired by the nuclear genome, then there would be an analogous energetic advantage to the host cell to utilize the acquired gene and delete the organellar gene.

Here, we analyze the relative cost of DNA synthesis and protein import over a broad range of plausible parameter spaces for eukaryotic cells that encompasses total cell protein content, organellar fraction (i.e., the fraction of the total number of protein molecules in a cell that is contained within the organelle), organellar genome copy number, organellar protein abundance, organellar protein import cost, organellar protein import efficiency, cell life span, and protein turnover rate. Through this, we reveal that for the vast majority of plausible parameter space for eukaryotic cells, it is energetically favorable to the cell to transfer organellar genes to the nuclear genome and re-import the proteins back to the organelle. We show that the interplay between per-cell organellar genome copy number and per-cell organellar protein abundance determines the magnitude of the energy saved such that it is only energy efficient for the cell to retain genes in the organellar genome if they encode proteins with very high abundance. Through analysis of the energy saved by endosymbiotic gene transfer in the context of total cellular energy budgets, we demonstrate that the net energetic advantage of endosymbiotic gene transfer is a significant proportion of total cell energy budgets and would thus confer a selectable energetic advantage to the cell. Collectively, these results reveal that enhanced energy efficiency has helped to shape the content and evolution of eukaryotic organellar and nuclear genomes.

## Results

### The cost to the cell to encode a gene in the organellar genome is higher than in the nuclear genome

**Fig. 1:**
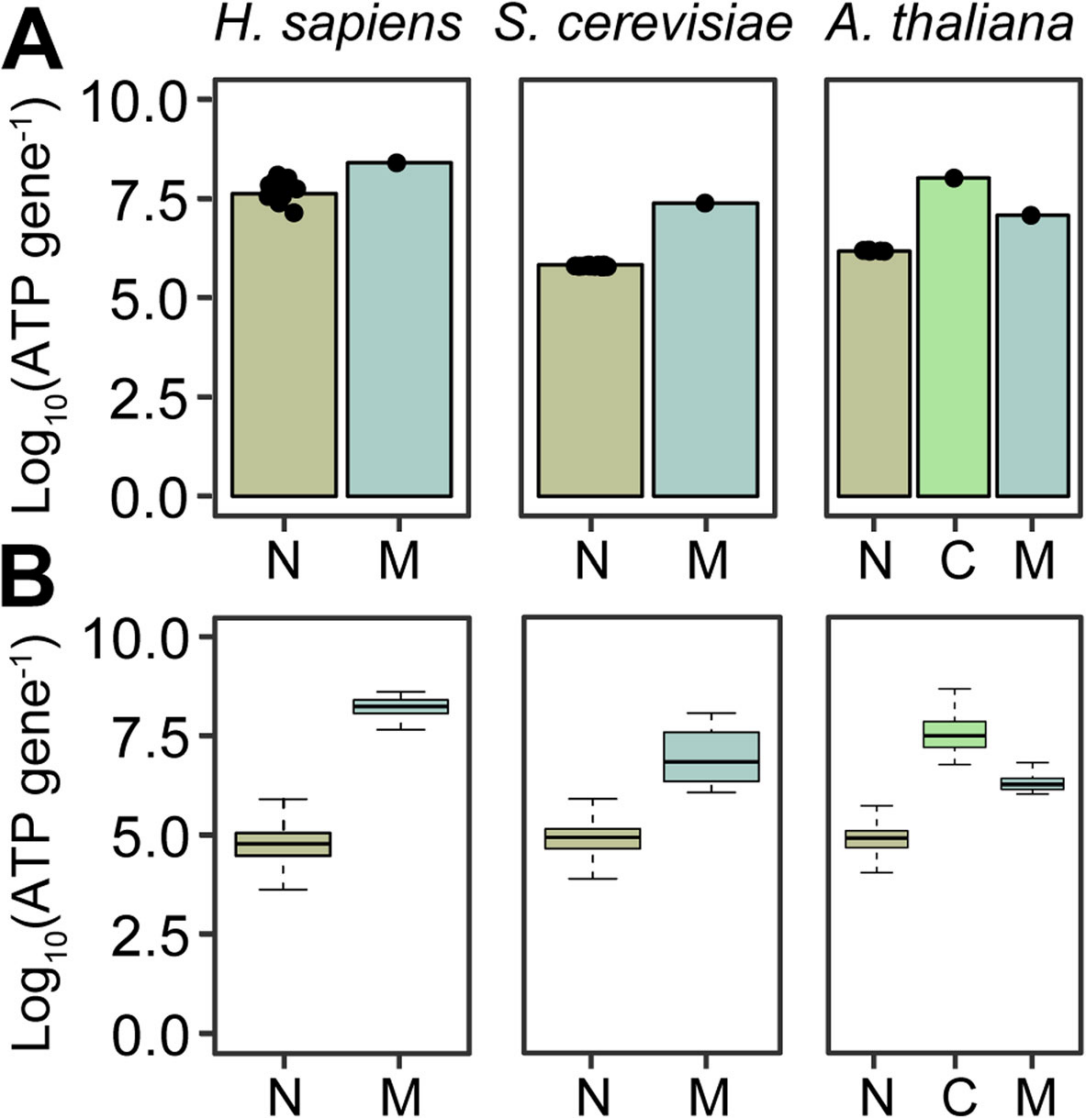
The per-cell biosynthetic cost of nuclear and organellar genes in three representative eukaryotes. **A** The ATP biosynthesis costs of nuclear (N), chloroplast (C), and mitochondrial (M) genes calculated as the cost of the chromosome divided by the number of genes contained within that chromosome. Nuclear chromosomes include the cost of nucleosomes, and organellar chromosomes only included the cost of the DNA. In the case of the nuclear genes, the height of the bar depicts the mean cost of all nuclear chromosomes with individual points showing all chromosomes overlaid on top of the bar plots. **B** The ATP biosynthesis cost of just the coding sequences of the genes. In both **A** and **B**, the costs were computed assuming a diploid nuclear genome, a per-cell mitochondrial genome copy number of 5000, 200, and 100 for the in *H. sapiens*, *S. cerevisiae*, and *A. thaliana*, respectively, and a per-cell chloroplast genome copy number of 1500 in *A. thaliana*

Eukaryotic cells possessing chloroplasts and/or mitochondria typically have a higher copy number of their organellar genomes than their nuclear genomes [68]. Accordingly, while a typical diploid cell will have two copies of every gene in the nuclear genome, the same cell will have hundreds to hundreds of thousands of copies of every organellar encoded gene [68]. This difference in per-cell genome copy number means that it costs the cell more DNA to encode a gene in the organellar genome than in the nuclear genome. To provide an illustration of this difference in cost, three model eukaryotes were selected with disparate genome sizes and organellar genome content which are representative of the diverse range of values that have been previously reported [68]. Here, the cost of encoding a gene in a nuclear or organellar genome was considered to be the ATP cost of the chromosome (organellar or nuclear) divided by the number of genes on that chromosome. This consideration was performed to account for the differences in organellar and nuclear genomes such as the presence of introns, structural elements (telomeres, centromeres, etc.), and regulatory elements. We also included the ATP cost of the requisite number of histone proteins contained in nucleosomes to compute the cost of encoding a gene in the nuclear genome. This revealed that the high per-cell organellar genome copy number meant that the ATP cost of encoding a gene in the organellar genome is on average one order of magnitude higher than the cost of encoding a gene in the nuclear genome (Fig. 1A). This difference in ATP cost is further enhanced if the cost of just the coding sequences (including nucleosomes but excluding introns and non-coding regions) is compared directly (Fig. 1B). This latter scenario is more similar to a recent endosymbiotic gene transfer that arrives in the nuclear genome without introns and acquires these over time [72]. As the three representative organisms shown here span the range of organellar genome copy numbers that have been observed in eukaryotes [68], it follows that the ATP cost to the cell of encoding a gene in the organellar genome is generally higher than the cost of encoding the same gene in the nuclear genome in eukaryotes. Consequently, for any organellar gene, the cell may be able to save resources by transferring that gene from the organellar genome to the nuclear genome or by acquiring a functionally equivalent gene through lateral gene transfer and deleting the organellar gene.

### The energy saved by encoding a gene in the nuclear genome instead of the organellar genome is sufficient to offset the cost of organellar protein import

**Fig. 2:**
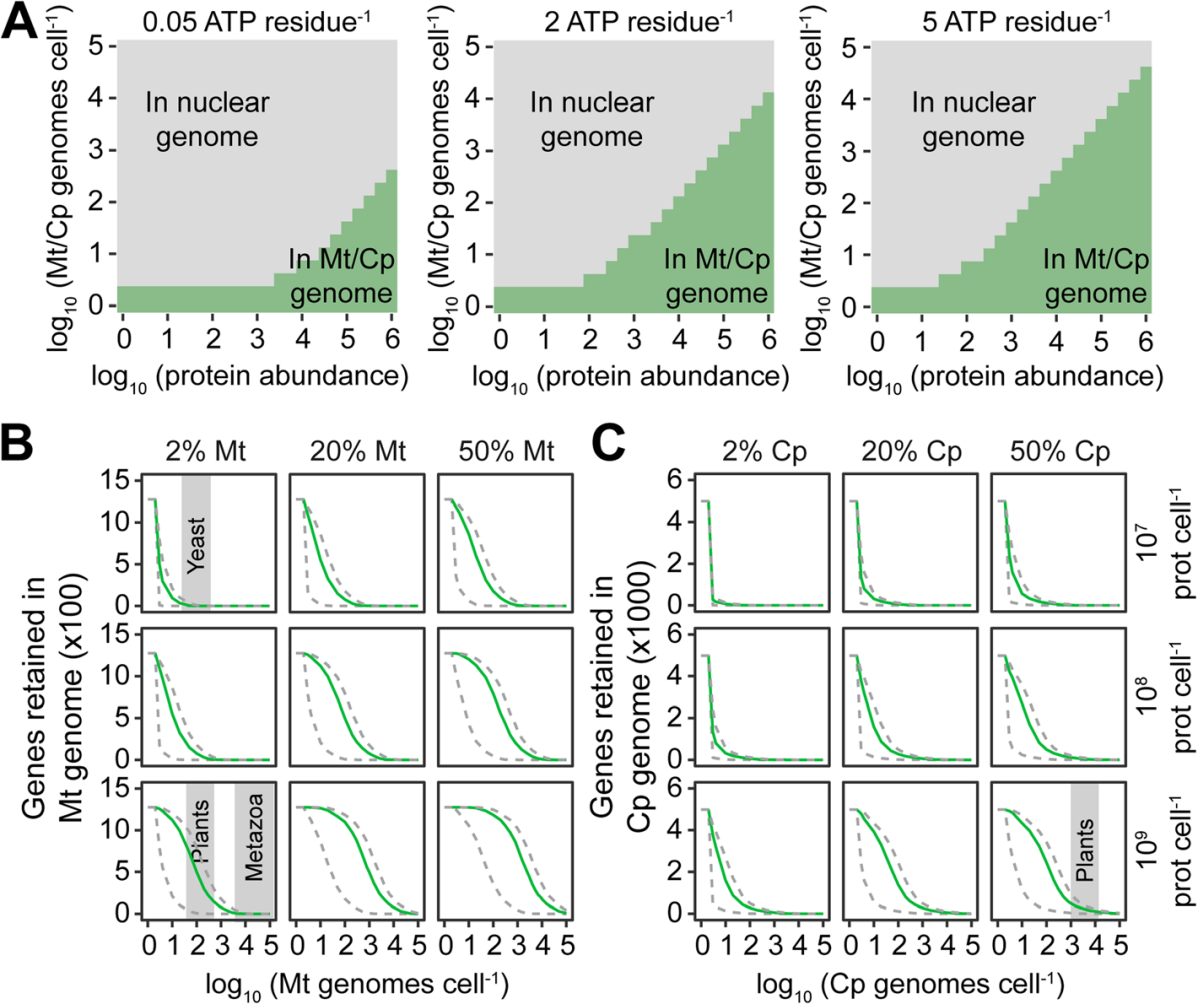
The minimum cost location to the cell of genes encoding an organellar localized proteins. **A** The minimum cost location of an organellar gene for a range of per-protein import costs, organellar genome copy numbers, and encoded protein abundance. The modeled per-residue protein import cost is shown above each plot. The gray-shaded fractions of the plots indicate the regions of parameter space where it is more energetically favorable to the cell to encode an organellar gene in the nuclear genome and import the requisite amount of protein. The green-shaded fractions of the plots indicate the regions of parameter space where it is more energetically favorable to the cell to encode the gene in the organellar genome. **B** The number of genes in the alphaproteobacterial (mitochondrial) genome for which it is more energetically favorable to the cell for the gene to be retained in the organellar genome. Green lines assume a per-residue protein import cost of 2 ATP per amino acid. Gray dashed lines indicate lower and upper cost bounds of 0.05 ATP and 5 ATP per residue, respectively. **C** As in **B** but for the cyanobacterial (chloroplast) genome. Gray-shaded areas on the plots are provided to indicate the organellar genome copy numbers of yeast, metazoan, and plant cells. Cp, chloroplast; Mt, mitochondrion

Although it is cheaper for the cell to encode a gene in the nuclear genome than the organellar genome, this direct cost comparison only considers the cost of DNA (and its associated proteins) and does not account for the additional cost that would be incurred should the product of a nuclear-encoded gene be required to function in the organelle. Such nuclear-encoded organelle-targeted proteins incur additional energetic costs to be translocated across the organellar membranes. Accordingly, to assess whether it is cheaper for the cell to encode an organelle-targeted protein in the nuclear or organellar genome, it is necessary to consider both the abundance of the encoded protein and the energetic cost of organellar protein import. Estimates for the energetic cost of mitochondrial or chloroplast protein import vary over two orders of magnitude from ~ 0.05 ATP per amino acid to 5 ATP per amino acid [73–75]. Thus, for the purposes of this study, the full range of estimates was considered and the range of conditions under which it is more energetically favorable to encode a gene in the organellar or nuclear genome was assessed. This analysis revealed that the higher the copy number of the organellar genome, the more energy that is saved by encoding the gene in the nuclear genome and thus the more protein that can be imported into the organelle while still reducing the overall energetic cost of the cell (Fig. 2A). As the per-cell gene copy number is the same for each gene encoded on the organellar genome, the possible energetic advantage to the cell arising from endosymbiotic gene transfer will vary between genes as a function of the required abundance of each encoded gene product. Furthermore, if the cell can function without the encoded gene product, then as organellar genome copy number increases the energetic incentive to discard the gene also increases. Thus, high organellar genome copy numbers provide an energetic incentive to either delete genes from the organellar genome or transfer them to the nuclear genome.

Given that the magnitude of the energetic advantage of endosymbiotic gene transfer is dependent on protein abundance, we sought to simulate the endosymbiotic genome reduction that would occur using realistic models of pre-mitochondrial and pre-chloroplast organellar progenitors. Here, the complete genomes with measured protein abundances for an alphaproteobacterium (*Bartonella henselae*) and a cyanobacterium (*Microcystis aeruginosa*) were chosen as models for the mitochondrial and chloroplast progenitors, respectively. In addition, a range of host cell size (i.e., host cell protein content) was considered such that it encompassed the majority of diversity exhibited by extant eukaryotes [76] and would thus likely also encompass the size range of the host cell that originally engulfed the organellar progenitors. This range extended from a small unicellular yeast-like cell (10^7^ proteins) to a large metazoan/plant cell (10^9^ proteins). Each of these cell types was then considered to allocate a realistic range of total cellular protein to mitochondria/chloroplasts representative of values observed in extant eukaryotic cells (Additional file 2: Table S1). For each set of conditions in this comprehensive parameter space, the energy liberated or incurred by endosymbiotic gene transfer was calculated for each organellar gene given its measured protein abundance [77] and a realistic range of protein import costs (including the total biosynthetic cost of the protein import machinery, see the “Methods” section). This revealed that for a broad range of estimates of cell size, organellar genome copy number, and organellar fraction (i.e., the fraction of the total number of protein molecules in a cell that are contained within the organelle), it is energetically favorable to the cell to transfer the majority of organellar genes to the nuclear genome and re-import the proteins back to the organelle (Fig. 2B, C). Only the proteins with the highest abundance, and thus which incur the largest import cost, are energetically favorable to be retained in the organellar genomes. This phenomenon was also observed even if extreme costs for protein import ten times those that have been measured are considered (Fig. S1). Thus, it is more energy efficient for a eukaryotic cell to position the majority of genes that encode organellar targeted proteins in the nuclear genome.

**Fig. 3:**
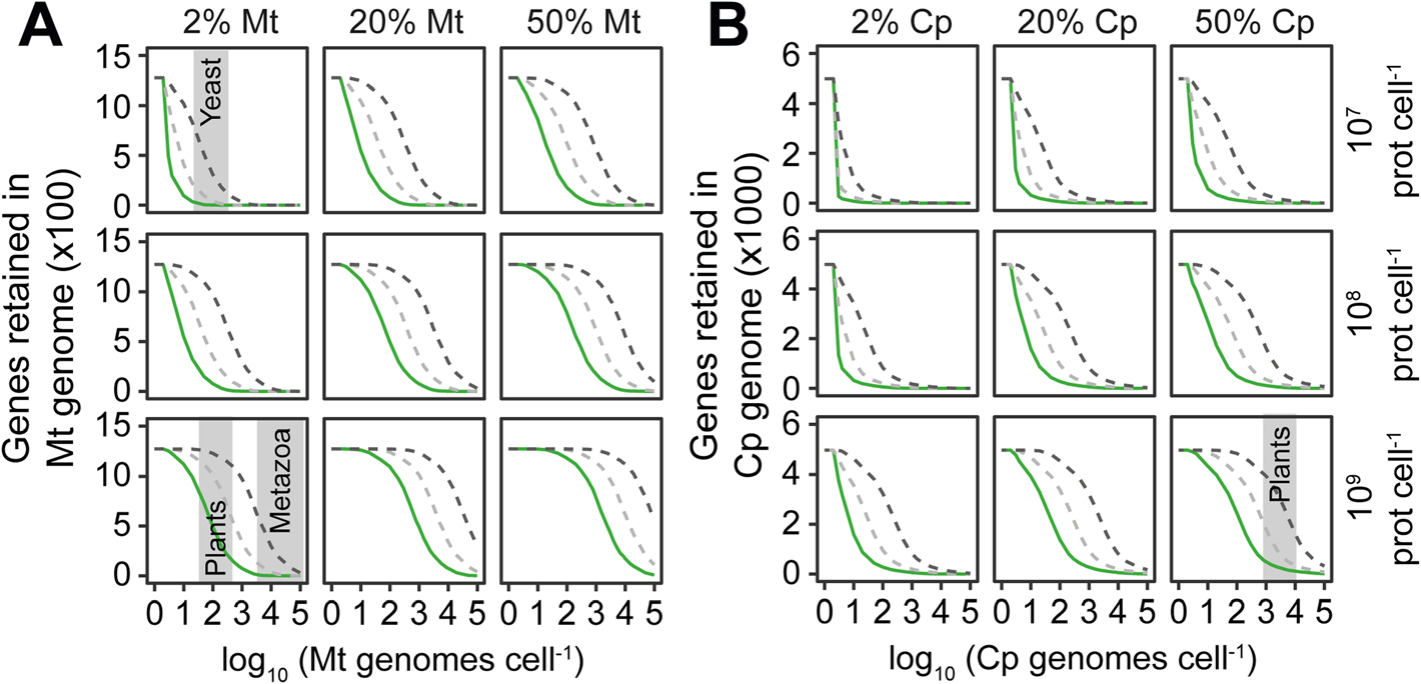
The impact of protein turnover on the energetic favorability of organellar gene retention. **A** The number of genes in the alphaproteobacterial (mitochondrial) genome for which it is more energetically favorable to the cell for the gene to be retained in the organellar genome. **C** As in **B** but for the cyanobacterial (chloroplast) genome. All lines assume a per-residue protein import cost of 2 ATP per amino acid. Green lines assume that protein turnover is mediated by dilution due to cell division. Light gray dashed lines assume that the complete pool of organellar proteins at the requisite abundance are replaced 5 times per cell doubling. Dark gray dashed lines assume that the complete pool of organellar proteins at the requisite abundance are replaced 50 times per cell doubling. Gray-shaded areas on the plots are provided for illustrative purposes to indicate the organellar genome copy numbers of yeast, metazoan, and plant cells. Cp, chloroplast; Mt, mitochondrion

The above analysis assumed that the total pool of cellular protein was replaced with each cell doubling. This assumption is consistent with the observations that protein turnover in eukaryotes (as in bacteria) is primarily mediated by dilution due to cell division [78–80], i.e., the vast majority of proteins have half-lives that are longer in duration than the doubling time of the cell, and thus protein turnover occurs through replicative dilution. However, a small population of proteins is turned over more than once per cell division cycle [78–80], and in multicellular organisms, there can be populations of cells with a low or negligible rate of cell division resulting in a higher rate of protein turnover per cell division. Similarly, some of the archaeal relatives of the last eukaryotic common ancestor have slow cell doubling rates and thus may have higher rates of protein turnover relative to cell doubling. Thus, to determine the impact of enhanced rates of protein turnover relative to cell doubling, the analysis above was repeated while increasing the rate of protein turnover from once per cell division cycle (i.e., dividing cells) to 50 times per cell doubling (i.e., a long-lived or non-dividing cell). Increasing the rate of protein turnover increases the total amount of protein that must be imported into the organelle (akin to an increase in absolute abundance of that protein) and thus leads to an increase in the number of proteins for which it is energetically favorable to retain their corresponding genes in the organellar genomes (Fig. 3A, B). However, even if it is assumed that the total pool of each organellar protein is turned over 50 times per cell doubling, it is still more energetically favorable to transfer the majority of organellar genes to the nuclear genome when the organellar genome copy number is high (Fig. 3A, B). Thus, in both dividing cells and in cells with higher rates of protein turnover relative to cell division, it is more energetically favorable to encode the majority of organellar targeted proteins in the nuclear genome.

### Proteins encoded by organellar genes have higher estimated ancestral abundance than those that have been lost or transferred to the nuclear genome

**Fig. 4:**
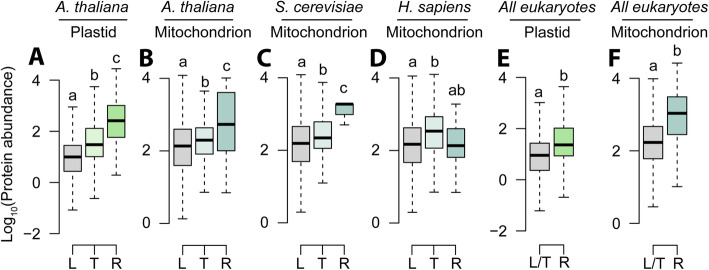
The abundance of proteins encoded by genes that have been lost, transferred to the nucleus, or retained in the organellar genome. **A** The abundance of proteins in the cyanobacterium *Microcystis aeruginosa* categorized according to whether their encoding genes have been lost, transferred to the *Arabidopsis thaliana* nuclear genome, or retained in the *Arabidopsis thaliana* chloroplast genome. **B** The abundance of proteins in the alphaproteobacterium *Bartonella henselae* categorized according to whether their encoding genes have been lost, transferred to the *Arabidopsis thaliana* nuclear genome, or retained in the *Arabidopsis thaliana* mitochondrial genome. **C**, **D** As in **B** but for *Saccharomyces cerevisiae* and *Homo sapiens*, respectively. **E** As in **A** but for all plastid genomes on NCBI. **F** As in **B** but for all mitochondrial genomes on NCBI. L, lost; T, transferred to the nuclear genome; R, retained in the organellar genome. Letters above the boxplots indicate whether there were significant differences between the means of different groups (*p* < 0.05) in the results of a one-way ANOVA with Tukey test for multiple comparisons

The analyses above predict that the proteins with the highest abundance, and thus those which incur the highest import costs, are those that are more likely to be retained in an organellar genome. While it is unknown how the abundance of proteins in organelles has changed throughout the evolution of the eukaryotes, it is possible to estimate what the profile of protein abundances may have looked like during the initial stages of this process by examining protein abundance in extant bacterial relatives of organelles [77]. Using these inferred ancestral protein abundance estimates, it is thus possible to ask whether those genes that are retained in the organellar genome are those that encode proteins with higher abundance than those that are lost or transferred to the nuclear genome. This revealed that the estimated abundance of the cohorts of proteins whose genes are retained in the chloroplast (Fig. 4A) and mitochondrial (Fig. 4B) genomes of *Arabidopsis thaliana* and the mitochondrial genome of *Saccharomyces cerevisiae* (Fig. 4C) is significantly higher than the estimated abundance of the cohorts of proteins that were either lost or transferred to the respective nuclear genomes. The estimated abundance of the cohort of proteins whose genes are retained in the mitochondrial genome of *Homo sapiens* was not significantly different from those that have been lost or transferred to the nuclear genome (Fig. 4D). To assess whether or not this elevated protein abundance was a general phenomenon, the full set of complete plastid and mitochondrial genomes were downloaded from NCBI, and the sets of genes present or absent from these genomes were analyzed. Here, the corresponding nuclear genomes were not available, so it was not possible to separately assess the estimated abundance proteins encoded by lost or putatively transferred genes, and thus, they were analyzed together. This analysis revealed that the estimated abundance of proteins encoded by genes found in the extant plastid (Fig. 4E) or mitochondrial (Fig. 4F) genomes in eukaryotes was significantly higher than those that have been lost or transferred to the nuclear genome. Thus, across all eukaryotes, the inferred ancestral abundance of proteins encoded by genes retained in organellar genomes is higher than those encoded by genes that were either lost or transferred to the nuclear genome.

### The energy saved by gene loss or endosymbiotic gene transfer is sufficient to produce a selectable advantage for the majority of genes

**Fig. 5:**
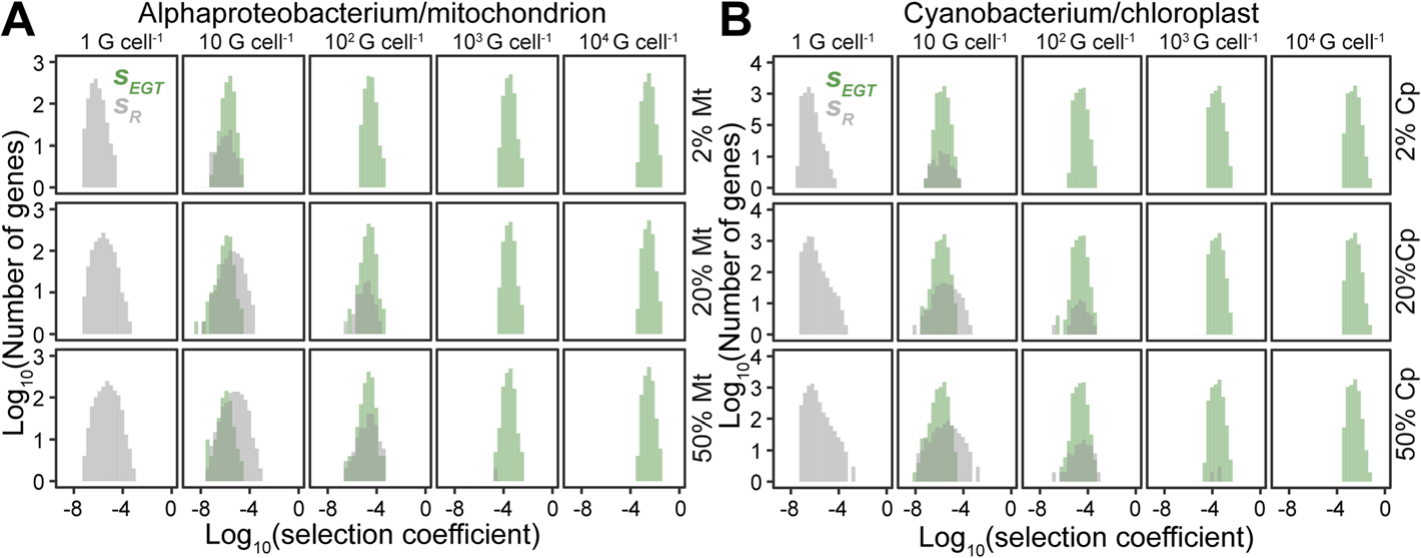
Selection coefficients for retention (*S*_*R*_, gray) or endosymbiotic gene transfer (*S*_*EGT*_, green) of all genes encoded in the example alphaproteobacterial and cyanobacterial genomes. Coefficients were computed accounting for protein abundance, host cell organellar fraction, organellar genome copy number per cell, and host cell energy consumption. The plots shown are for a simulated host cell comprising 1 × 10^7^ proteins and a protein import cost of 2 ATP per residue, plots for other host cell protein contents and protein import costs are provided in Additional file 1: Fig. S2-S7. **A** Selection coefficients of all genes encoded in the alphaproteobacterium genome. **B** Selection coefficients for all genes encoded in the cyanobacterial genome. *S*_*R*_ and *S*_*EGT*_ have opposite signs (see the “Methods” section). To simplify the display and enable direct comparison, the absolute value of the selection coefficients of each gene is plotted, and green shading is used to indicate genes in the *S*_*EGT*_ fraction and gray shading indicates genes in the *S*_*R*_ fraction of the genome. Mt, mitochondrion; Cp, chloroplast; G, genomes

Although gene loss or endosymbiotic gene transfer can save energy, the question arises as to whether this energy saving would be sufficient to confer a selectable advantage for the cell. To estimate this, the energy liberated by endosymbiotic gene transfer of each gene encoded in the ancestral pre-organellar genomes was evaluated as a proportion of the total energy required to replicate the cell. As above, this analysis was conducted for a broad range of host cell size, organellar fraction, endosymbiont/organellar genome copy number, and protein import cost that is representative of a broad range of eukaryotic cells (Fig. 5A, B; Figs. S2–S7). This revealed that for even modest per-cell endosymbiont genome copy numbers (~ 100 copies per cell), the proportion of the total cell energy budget that could be saved for an individual gene transfer event (or equivalent functional lateral complementation) is sufficient that it would confer a selectable advantage. If the energetic advantage is considered to be a direct fitness advantage then the selection coefficients for the transfer of the majority of individual endosymbiont genes are ~ 1 × 10^−5^ (Fig. 5; Figs. S2–S7). This is ~ 1000 times stronger than the selection coefficient acting against disfavored synonymous codons [81]. Moreover, for high per-cell endosymbiont genome copy numbers (~ 1000 genome copies per cell), these selection coefficients are proportionally larger (~ 1 × 10^−4^), equivalent to approximately 1/10th the strength of the selection that caused the allele conferring lactose tolerance to rapidly sweep through human populations in ~ 500 generations [82]. In contrast, selection coefficients for retention of genes in the organellar genome generally only occur when organellar genome copy numbers are low, and/or when large proportions of cellular resources are invested in the organelle (Fig. 5A, B; Figs. S2–S7). Consistent with the analysis of protein turnover relative to cell doubling time (Fig. 3), these results are recovered even for cells with ten times the cell doubling time considered here (Figs. S8–S13). Thus, over a broad range of host cell sizes, organellar genome copy numbers, organellar fractions, and per-protein ATP import costs, protein turn-over rates, and cell doubling times endosymbiotic gene transfer of the majority of genes is sufficiently energetically advantageous that any such transfer events, if they occurred, would confer an energetic advantage to the cell and have the potential to rapidly reach fixation (Fig. S14). Thus, endosymbiotic gene transfer of the majority of organellar genes is advantageous to eukaryotic cells.

## Discussion

The endosymbiosis of the bacterial progenitors of the mitochondrion and the chloroplast are landmark events in the evolution eukaryotes. Following these endosymbioses, there was a dramatic reduction in the gene content of the organellar genomes such that they now harbor fewer than 5% of the genes found in their free-living bacterial relatives. Some of these genes have been discarded, but many have been transferred to the nuclear genome and their products (proteins) are imported back into the organelle where they function. The reason why these organelles have transferred their genes to the nucleus is a long-standing unanswered question in evolutionary biology. Here, we show, through extensive simulation of plausible parameter spaces for eukaryotic cells, that there are energy incentives for gene loss and for endosymbiotic gene transfer from organellar genomes. We show that these energy incentives are dependent on the abundance of the encoded gene product, with a trade-off between per-cell organellar genome copy number and protein abundance determining the magnitude and direction of the energy incentive. We further show that these energy incentives can be sufficient to produce a selectable advantage to the host cell for both endosymbiotic gene transfer and retention of genes in the organellar genomes. Thus, the economics of protein production and transport plays a role in determining whether genes are lost, retained, or transferred from organellar genomes.

Although this study reveals that the energy efficiency of protein production can provide a driver for the location of an organellar gene, it is not proposed that it is the only factor that influences this process. Instead, a large cohort of factors including the requirement for organellar-mediated RNA editing, protein chaperones, protein folding, post-translational modifications, escaping mutation hazard, Muller’s rachet, enhanced nuclear control, the requirement for redox regulation of gene expression, and drift will act antagonistically or synergistically with energetic incentives described here to influence the set of genes that are retained in, lost, or transferred from, the organellar genomes. The study presented here simply reveals that energy efficiency is a previously overlooked factor that has likely played a role in shaping the evolution organellar/nuclear genomes. Moreover, the work presented here is in agreement, and is synergistic, with previous hypotheses that have suggested that the reason for retaining genes in organellar genomes is that there is a selectable advantage to do so. Specifically, the CoRR hypothesis [42, 63–65] posits that genes are retained in organellar genomes as it is advantageous for the cell to be able to control gene expression (and the gene products that are made) in immediate and direct response to the redox state of the organelle. These redox-regulated genes are also required in very high abundance within the organelle, and thus, the selection on energetic incentives acts in the same direction as selection for maintaining tight redox regulation. Stochastic models of populations of cells in which endosymbiotic gene transfer (or functionally equivalent lateral gene transfer) is occurring may provide insight into the synergy and conflict between this diverse set of factors, and their relative contribution to the evolution of organellar genomes. 

It is noteworthy in these contexts that if the protein encoded by the endosymbiont gene can provide its function outside of the endosymbiont (e.g., by catalyzing a reaction that could occur equally well in the cytosol of the host as in the endosymbiont), then the energetic advantage of gene transfer to the nuclear genome is further enhanced, as the cost of protein import is not incurred. Similarly, although gene loss has been proposed to be mediated predominantly by mutation pressure and drift [20], the elevated per-cell endosymbiont genome copy number also provides a substantial energetic reward to the host cell for complete gene loss as neither the costs of encoding the gene or producing its product are incurred. Thus, high organellar genome copy number provides an energetic incentive for the cell to delete endosymbiont genes or transfer them to the nuclear genome.

While the analysis presented here focussed on the energetic cost measured in ATP so that the cost of protein import and the cost of biosynthesis of DNA could be evaluated on a common basis, endosymbiotic gene transfer also results in changes in the elemental requirements of a cell. Specifically, as the monophosphate nucleotides that constitute DNA are composed of carbon (A = 10, C = 9, G = 10, T = 10), nitrogen (A = 5, C = 3, G = 5, T = 2), and phosphorous (A = 1, C = 1, G = 1, T = 1) atoms, endosymbiotic gene transfer can also result in substantial savings of these resources (Fig. S15). Thus, if organisms encounter carbon, nitrogen, or phosphorous limitation in their diet and environment, then the advantage of endosymbiotic gene transfer to the cell will be further enhanced.

**Fig. 6:**
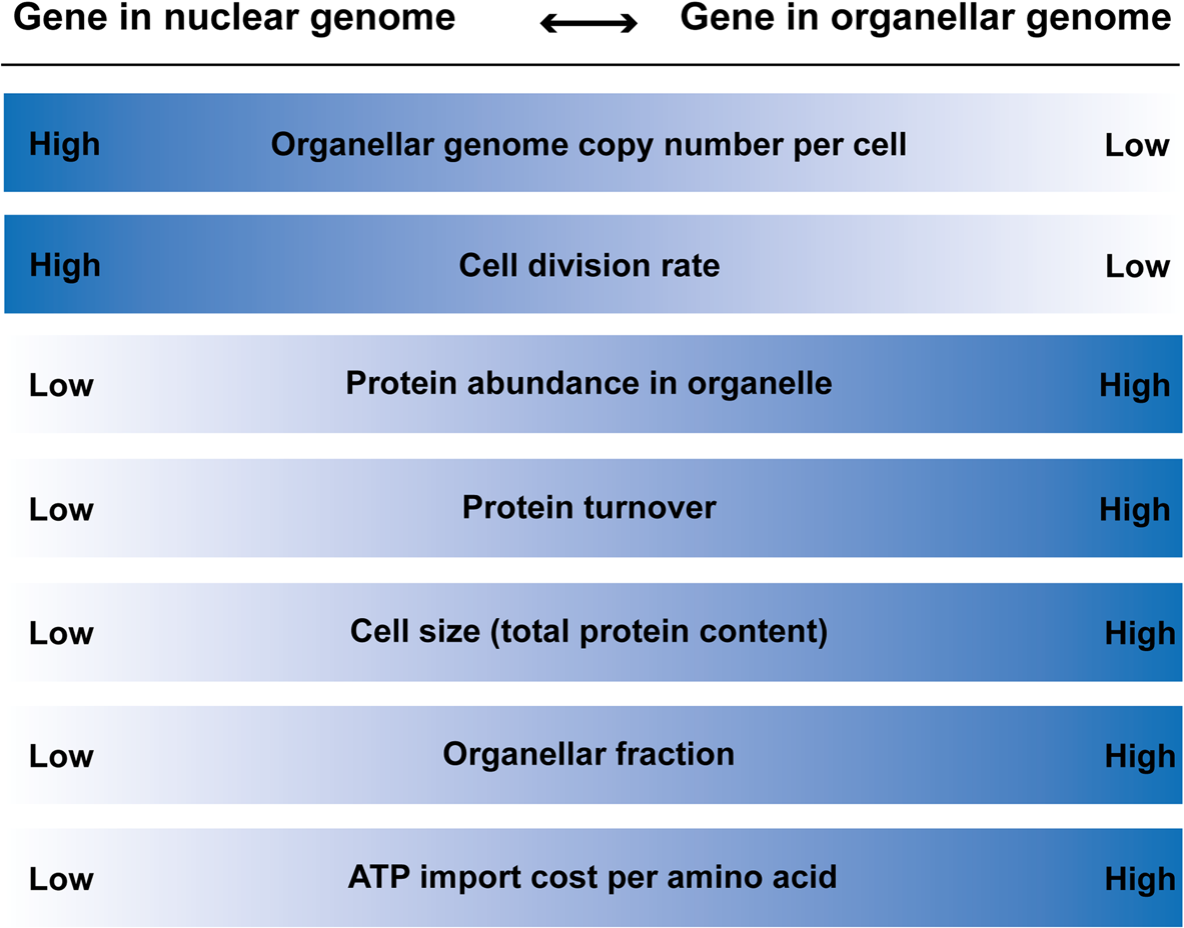
The competing factors that influence the energetically optimal location of a gene encoding an organellar targeted protein. Many of these factors are linked (e.g., protein abundance in organelle and organellar fraction, or cell division rate and protein turn-over) and are provided here for completion

The analysis presented here shows that a broad range of cell sizes and resource allocations that endosymbiotic gene transfer of the majority of organellar genes is energetically favorable and thus advantageous to the cell. However, it also showed that retention of genes in the organellar genomes is energetically favorable under conditions where the encoded organellar protein is required in very high abundance and/or the copy number of the organellar genome is low. Other interlinked competing factors that influence the energetically optimal location of a gene are shown in Fig. 6. Each of these factors interacts to influence the cost to the cell for encoding a gene in the nuclear or organellar genome. This is important, as while we do not know precisely what the cells that engulfed the progenitors of the mitochondrion or the chloroplast looked like (as only extant derivatives survive), it is safe to assume that cell size and investment in organelles has altered since these primary endosymbioses first occurred. Accordingly, the selective advantage (or disadvantage) of transfer of any given gene is transient and will have varied during the radiation of the eukaryotes as factors such as cell size and organellar volume evolved and changed in disparate eukaryotic lineages. This coupled with the lack of an organellar protein export system (i.e., from the organelle to the host cytosol) and the presence (and acquisition) of introns in nuclear-encoded genes [83] means that it is more difficult for endosymbiotic gene transfer to operate in the reverse direction (i.e., from the nucleus to organelle). Similarly, eukaryotic cells can typically tolerate the loss of one or more chloroplasts [84] or mitochondria [85] from a cell without the concomitant death of the cell, the disruption of these organelles is thought to be a major route through which DNA from organelles enters the nucleus and can thus be incorporated into the nuclear genome. The converse process (i.e., the loss of the nucleus) is terminal to the cell and is thought to be a major reason why endosymbiotic gene transfer operates in one direction only. Collectively, these factors would create ratchet-like effect trapping genes in the nuclear genome even if subsequent changes in cell size and organellar fraction means that it became energetically advantageous to return the gene to the organelle later in the evolution. Thus, current organellar and nuclear gene contents predominantly reflect past pressures to delete organellar genes or transfer them to the nuclear genome.

## Conclusion

Endosymbiotic gene loss and gene transfer are a recurring theme in the evolution of the eukaryotic tree of life. The discovery that endosymbiotic gene transfer (or equivalent functional lateral complementation) can provide an energetic advantage to the cell for loss, retention, or transfer of organellar genes to the nuclear genome uncovers a novel process that has helped shape the content and evolution of eukaryotic genomes.

## Methods

### Data sources

The *Arabidopsis thaliana* genome sequence and the corresponding set of representative gene models were downloaded from Phytozome V13 [86]. The human genome sequence and gene models from assembly version GRCh38.p13 (GCA_000001405.28), the *Bartonella henselae* genome sequence and gene models from assembly version ASM4670v1, and the *Microcystis aeruginosa* NIES-843 genome sequence and gene models from assembly version ASM1062v1 were each downloaded from Ensembl [87]. The *Saccharomyces cerevisiae* sequence and gene models from assembly version R64-2-1_20150113 were downloaded from the *Saccharomyces* Genome Database [88]. Protein abundance data for all species were obtained from PAXdb v4.1 [77].

### Constants used to evaluate the per cell ATP costs of genes and chromosomes

The ATP biosynthesis cost of nucleotides and amino acids was obtained from [89] and [71] and are provided in Additional file 3: Table S2. The *Homo sapiens* mitochondrial genome copy number of 5000 was obtained from [68]. The *Saccharomyces cerevisiae* mitochondrial genome copy number of 200 was obtained from [90]. The *Arabidopsis thaliana* chloroplast genome copy number of 1500 was obtained from [91], and the *Arabidopsis thaliana* mitochondrial genome copy number of 100 was obtained from [68].

For genes in nuclear chromosomes, the cost of DNA was calculated to include the cost of nucleosomes with one histone octamer comprising two copies each of the histone proteins H2A, H2B, H3, and H4 every 180 bp (147 bp for the two turns of DNA around the histone octamer and 33 bp for the spacer) [71]. For organellar chromosomes, there are no histones/nucleosomes, and thus, the biosynthetic cost of genes in organellar chromosomes was calculated as the cost of the DNA divided by the number of genes on the chromosome (Additional file 4: Table S3). Although there are no histone protein equivalents in that organellar genomes, it should be noted that there are some nuclear-encoded proteins that are known to bind mitochondrial or chloroplast DNA. The costs associated with these proteins have not been included here as their function in packaging DNA is unknown and their density within the organellar genome is also unknown, and it is thus difficult to estimate their required abundance. However, the inclusion of the production and import costs of these proteins would further increase the cost of encoding a gene in the organellar genome and would accentuate the differences shown in this study.

The average gene length used for the simulation study in Fig. 2 was obtained by computing the average gene length across the two bacterial genomes used in this study, *Bartonella henselae* ASM4670v1 and Microcystis *aeruginosa* NIES-843.

### Calculating protein import costs

Although the molecular mechanisms of mitochondrial and chloroplast protein import differ [92–94], they share many commonalities including the requirement for energy in the form of nucleoside triphosphate hydrolysis [95]. The energetic cost of mitochondrial or chloroplast protein import is difficult to measure directly, and accordingly, estimates vary over two orders of magnitude from ~ 0.05 ATP per amino acid to 5 ATP per amino acid [73–75]. Thus, for the purposes of this study, the full range of estimates was considered in all simulations when evaluating the import cost of organellar targeted proteins encoded by nuclear genes.

The cost of the biosynthesis of the protein import machinery (i.e., the TOC/TIC or TOM/TIM complexes, Additional file 5: Table S4) was also included in the per protein import costs calculated in this study. For *Arabidopsis thaliana*, if the total ATP biosynthesis cost of all TOC/TIC complex proteins in the cell (i.e., the full biosynthesis cost of all the amino acids of all the proteins at their measured abundance in the cell) is distributed equally among all of the proteins that are imported into the chloroplast, then it would add an additional 0.2 ATP per residue imported (Additional file 6: Table S5). Similarly, if the total ATP biosynthesis cost of all TOM/TIM proteins in the cell in *Homo sapiens*, *Saccharomyces cerevisiae*, and *Arabidopsis thaliana* is distributed equally among all of the proteins that are imported into the mitochondrion in those species, then it would add an additional 0.2 ATP, 0.7 ATP, and 0.2 ATP per residue imported, respectively (Additional file 6: Table S5). In all cases, protein abundance was calculated using measured protein abundance estimates for each species obtained from PAXdb 4.0 [77], assuming a total cell protein content of 1 × 10^9^ proteins for a human cell, 1 × 10^7^ proteins for a yeast cell, and 2.5 × 10^10^ proteins for an *Arabidopsis thaliana* cell. As we modeled ATP import costs from 0.05 ATP to 50 ATP per residue, the cost of the import machinery was considered to be included within the bounds considered in this analysis.

### Evaluating the proportion of the total proteome invested in organelles

To provide estimates of the fraction of cellular protein resources invested in organellar proteomes, the complete predicted proteomes and corresponding protein abundances were quantified. Organellar targeting was predicted using TargetP-2.0 [96], and protein abundance estimates were obtained from PAXdb 4.0 [77]. The proportion of cellular resources are provided in Additional file 2: Table S1 and were used to provide the indicative regions or parameter space occupied by metazoa, yeast, and plants shown in Fig. 2B, C. Specifically, ~ 5% of total cellular protein is contained within mitochondria in *H. sapiens*, *S. cerevisiae*, and *A. thaliana*, and ~ 50% of total cellular protein is contained within chloroplasts in *A. thaliana*.

### Calculating the free energy of endosymbiotic gene transfer

The free energy of endosymbiotic gene transfer (*ΔE*_*EGT*_) is here defined as the difference in energy cost to the cell to encode a given gene in the organellar genome and the cost to encode the same gene in the nuclear genome and import the requisite amount of gene product into to the organelle. *ΔE*_*EGT*_ is evaluated as the difference in ATP biosynthesis cost required to encode a gene (*ΔD*) in the endosymbiont genome (*D*_end_) and the nuclear genome (*D*_nuc_) minus the difference in ATP biosynthesis cost required to produce the protein (*ΔP*) in the organelle (*P*_end_) vs in the cytosol (*P*_cyt_) and ATP cost to import the protein into the organelle (*P*_import_). Such that:

1 $$ \varDelta {E}_{EGT}=\varDelta D-\varDelta P $$

where

2 $$ \varDelta D={D}_{\mathrm{end}}-{D}_{\mathrm{nuc}} $$

and

3 $$ \varDelta P={P}_{\mathrm{end}}-{P}_{\mathrm{cyt}}-{P}_{\mathrm{import}} $$

Thus, *ΔE*_*EGT*_ can be positive or negative depending on the cost associated with each parameter. The energetic cost of producing a protein in the endosymbiont and in the cytosol is assumed to be equal, and thus:

4 $$ \varDelta P={P}_{\mathrm{import}} $$

*P*_import_ is evaluated as the product of the length of the amino acid sequence (*L*_prot_), the ATP cost of importing a single residue from the contiguous polypeptide chain of that protein (*C*_import_), and the number of copies of that protein contained within the cell that must be imported (*N*_*p*_) such that:

5 $$ \varDelta P={P}_{\mathrm{import}}={L}_{\mathrm{prot}}{C}_{\mathrm{import}}{N}_p $$

Measured estimates of *C*_import_ range from ~ 0.05 ATP per amino acid to 5 ATP per amino acid [73–75]. For the purposes of this study, we used these measured ranges and also modeled a *C*_import_ up to 10 times higher than any measured estimate, i.e., from 0.05 ATP to 50 ATP.

Both *D*_end_ and *D*_nuc_ are evaluated as the product of the ATP biosynthesis cost of the double-stranded DNA (*A*_DNA_) that comprises the gene under consideration and the copy number (*C*) of the genome in the cell such that:

6 $$ {D}_{\mathrm{end}}={A}_{\mathrm{DNA}}{C}_{\mathrm{end}} $$

And

7 $$ {D}_{\mathrm{nuc}}={A}_{\mathrm{DNA}}{C}_{\mathrm{nuc}} $$

Such that:

8 $$ \varDelta D={A}_{\mathrm{DNA}}\left({C}_{\mathrm{end}}-{C}_{\mathrm{nuc}}\right) $$

where *C*_end_ and *C*_nuc_ are the per-cell copy number of the endosymbiont and nuclear genomes, respectively, and the ATP biosynthesis cost for the complete biosynthesis of an A:T base pair and a G:C base pair is 40.55 ATP and 40.14 ATP, respectively [89]. Thus:

9 $$ \varDelta {E}_{EGT}={A}_{\mathrm{DNA}}\left({C}_{\mathrm{end}}-{C}_{\mathrm{nuc}}\right)-{L}_{\mathrm{prot}}{C}_{\mathrm{import}}{N}_p $$

where positive values of *ΔE*_*EGT*_ correspond to genes for which it is more energetically favorable to be encoded in the nuclear genome, and negative values correspond to genes for which it is more energetically favorable to be encoded in the endosymbiont genome. Other studies have used slightly higher estimates (~ 50 ATP per nucleotide) for the biosynthesis cost of nucleotides [71, 97]. However, as this value is always used in the product with the difference in per-cell copy number of the endosymbiotic and nuclear genomes [8, 9], this would have a marginal effect on the results of the models. This is because the difference in copy number ranges over 5 orders of magnitude while the difference in the estimates of nucleotide biosynthesis cost varies by 20%.

### Simulating endosymbiotic gene transfer of mitochondrial and chloroplast genes

The complete genomes with measured protein abundances for an alphaproteobacterium (*Bartonella henselae*) and a cyanobacterium (*Microcystis aeruginosa*) were selected to serve as models for an ancestral mitochondrion and cyanobacterium, respectively. To account for uncertainty in the size and complexity of the ancestral pre-mitochondrial and pre-chloroplast host cells, a range of potential ancestral cells was considered to be engulfed by a range of different host cells with protein contents representative of the diversity of extant eukaryotic cells [76]. Specifically, the size of the host cell ranged from a small unicellular yeast-like cell (10^7^ proteins) to a medium-sized unicellular algal-like cell (10^8^ proteins) to a typical metazoan/plant cell (10^9^ proteins). Each of these host cell types was then considered to allocate a realistic range of total cellular protein to mitochondria/chloroplasts typical of eukaryotic cells (i.e., ~ 2% for yeast [98], ~ 20% for metazoan cells [99], and ~ 50% of the non-vacuolar volume of plant cells [100]). It is not important whether the organellar fraction of the cell is composed of a single large organelle or multiple smaller organelles as all costs, abundances, and copy numbers are evaluated at a per-cell level. For each simulated cell, *ΔE*_*EGT*_ was evaluated for each gene in the endosymbiont genome using real protein abundance data [77] for a realistic range of endosymbiont genome copy numbers using Eq. 9. In all cases, the host cell was assumed to be diploid. The simulations were repeated for three different per-residue protein import costs (0.05 ATP, 2 ATP, and 5 ATP per residue). The number of genes where *ΔE*_*EGT*_ was positive was recorded as these genes comprise the cohort that is energetically favorable to be encoded in the nuclear genome.

### Estimating the strength of selection acting on endosymbiotic gene transfer

To model the proportion of energy that would be saved by an individual endosymbiotic gene transfer event, a number of assumptions were made. It was assumed that the ancestral host cell had a cell size that is within the range of extant eukaryotes (i.e., between 1 × 10^7^ proteins per cell and 1 × 10^9^ proteins per cell). It was assumed that the endosymbiont occupied a fraction of the total cell proteome that is within the range exhibited by most eukaryotes today (2 to 50% of total cellular protein is located within the endosymbiont under consideration). It was assumed that endosymbiont genome copy number ranged between 1 copy per cell (as it most likely started out with a single copy) and 10,000 copies per cell.

We assumed an ancestral host cell with a 24-h doubling time such that all genomes and proteins are produced in the required abundance every 24-h period. As previously defined [71], the energy required for cell growth was modeled as:

10 $$ {C}_r=26.92{V}^{0.97} $$

In addition, all cells, irrespective of whether they are bacterial or eukaryotic, consume ATP (*C*_*m*_) in proportion to their cell volume (*V*) at approximately the rate of:

11 $$ {C}_m=0.39{V}^{0.88} $$

where *C*_*m*_ is in units of 10^9^ molecules of ATP cell^−1^ h^−1^, and V is in units of μm^3^ [71]. Thus, the total energy (*E*_*R*_) needed to replicate a cell was considered to be:

12 $$ {E}_R={C}_r+24\ {C}_m $$

The proportional energetic advantage or disadvantage (*E*_*A/D*_) to the host cell from the endosymbiotic gene transfer of a given gene is evaluated as the free energy of endosymbiotic gene transfer divided by the total amount of energy consumed by the cell during its 24-h life cycle.

13 $$ {\mathrm{E}}_{A/D}=\frac{\varDelta {E}_{EGT}}{E_R} $$

Given that *E*_*A/D*_ describes the proportional energetic advantage or disadvantage a cell has from a given endosymbiotic gene transfer event *E*_*A/D*_ can be used directly as selection coefficient (*s*) to evaluate the strength of selection acting on the endosymbiotic gene transfer of a given gene, such that:

14 $$ s={E}_{A/D} $$

As *ΔE*_*EGT*_ can be positive or negative as described above, *s* is therefore also positive or negative depending on the endosymbiont genome copy number, endosymbiont fraction, host cell protein content, the abundance of the protein that must be imported, and the ATP cost of protein import. When *s* is less than 0, the absolute value of *s* is taken to be the selection coefficient for retention of a gene in the endosymbiont genome (*S*_*R*_); when *s* is greater than 0, the value of *s* is taken to be the selection coefficient for endosymbiotic gene transfer to the nucleus (*S*_*EGT*_).

### Estimating time to fixation

Fixation times for endosymbiotic gene transfer events for a range of observed selection coefficients from 1 × 10^−5^ to 1 × 10^−2^ were estimated using a Wright-Fisher model with selection and drift [101, 102] implemented in a simple evolutionary dynamics simulation [103]. The effective population size for these simulations was set as 1 × 10^7^, as is representative of unicellular eukaryotes [104], and multicellularity in eukaryotes is not thought to have evolved until after the endosymbiosis of either the mitochondrion or the chloroplast.

## Supplementary Information

**Additional file 1.** This file contains the 15 supplemental figures and their associated legends.

**Additional file 2: Table S1.** Proportion of total protein allocated to organelles.

**Additional file 3: Table S2.** The ATP biosynthesis costs of nucleotides and amino acids used in this study.

**Additional file 4: Table S3.** The cost of encoding genes in organellar vs nuclear chromosomes.

**Additional file 5: Table S4.** The protein components of the organellar protein import complexes.

**Additional file 6: Table S5.** The additional per-residue costs of including the protein import machinery.

**Additional file 7.** Review history

## Data Availability

The *Arabidopsis thaliana* genome sequence and the corresponding set of representative gene models were downloaded from Phytozome V13 [86]. The human genome sequence and gene models from assembly version GRCh38.p13 (GCA_000001405.28), the *Bartonella henselae* genome sequence and gene models from assembly version ASM4670v1, and the *Microcystis aeruginosa* NIES-843 genome sequence and gene models from assembly version ASM1062v1 were each downloaded from Ensembl [87]. The *Saccharomyces cerevisiae* sequence and gene models from assembly version R64-2-1_20150113 were downloaded from the *Saccharomyces* Genome Database [88]. Protein abundance data for all species were obtained from PAXdb v4.1 [77].
