# Supplementary material for: The economics of organellar gene loss and endosymbiotic gene transfer
Source: Genome Biol. 2021 Dec 20;22:345. doi: 10.1186/s13059-021-02567-w (PMC8686548; doi:10.1186/s13059-021-02567-w)
Supplement: Supplementary file 1 — Additional file 1. This file contains the 15 supplemental figures and their associated legends. [file 13059_2021_2567_MOESM1_ESM.docx]

# Supplemental Figures

## Fig. S1

**
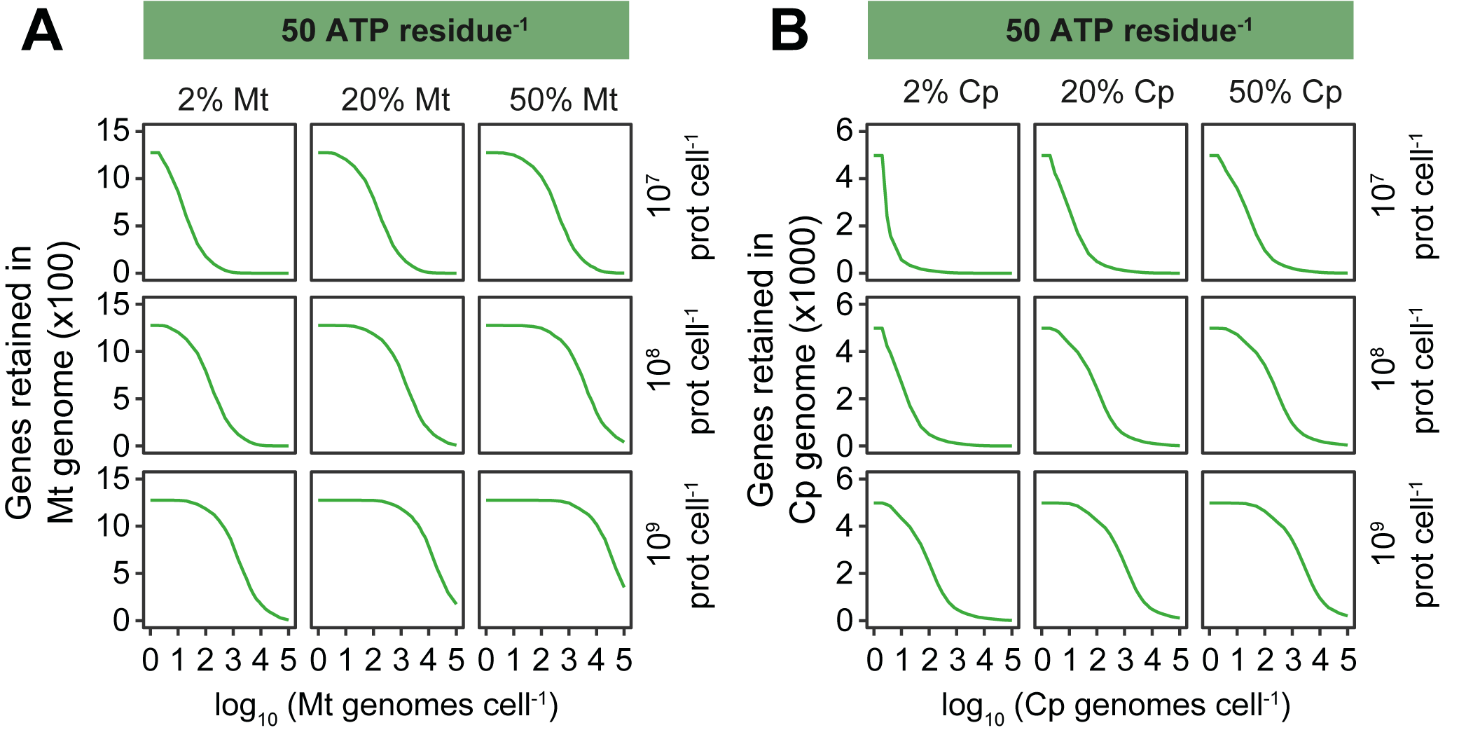
**

**Supplemental Figure S1.** The effect of assuming an ATP import cost 10 fold higher than the upper estimates of import cost measured in cells. **A)** The number of genes in the alphaproteobacterial (mitochondrial) genome for which it is more energetically favourable to the cell for the gene to be retained in the organellar genome. Green lines assume a per-residue protein import cost of 50 ATP per amino acid. **B**) As in A but for the cyanobacterial (chloroplast) genome.

## Fig. S2

**
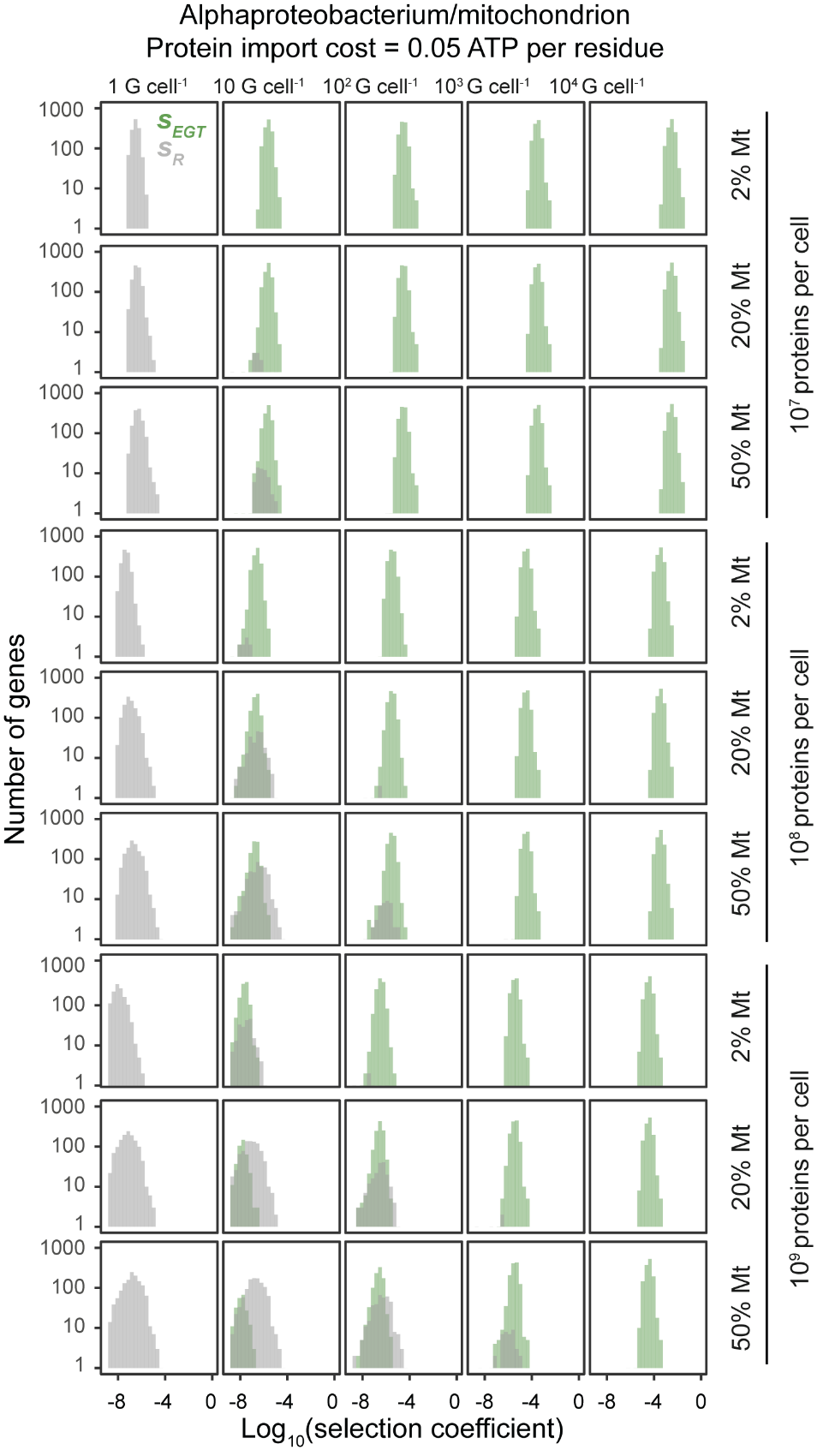
**

**Supplemental Figure S2** The selection coefficients for endosymbiotic gene transfer of alphaproteobacterial genes for hypothetical cell with a cell doubling time of 24 hours as a function of host cell size, host cell mitochondrial fraction and mitochondrial genome copy number per cell for a protein import cost of 0.05 ATP per residue. Histograms depict the selection coefficients for all genes in the endosymbiont genome. *S_R_* and *S_EGT_* have opposite signs (see methods), however to simplify the display and enable comparison the absolute value of the selection coefficients of each gene plotted.

## Fig. S3


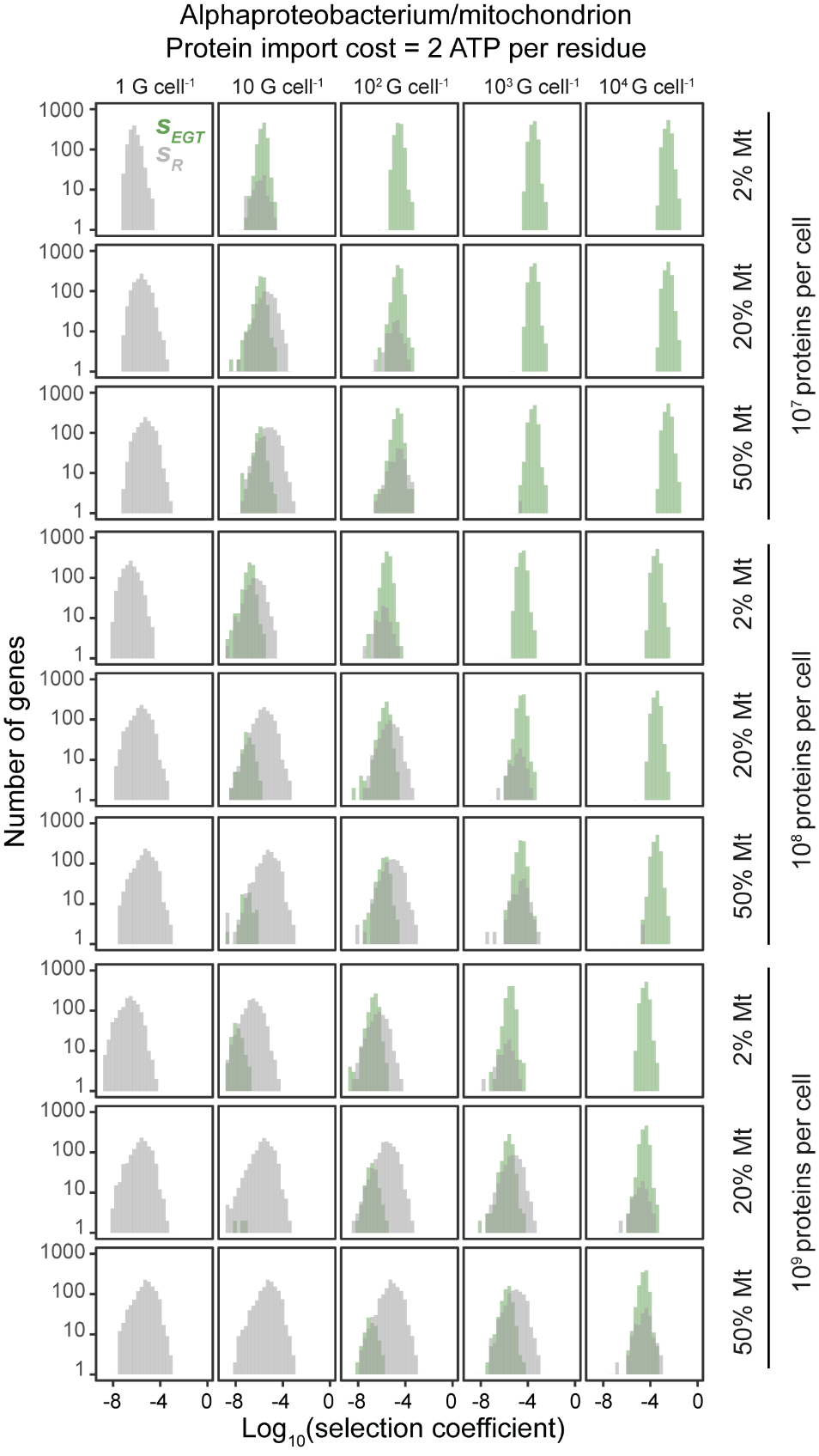


**Fig. S3.** The selection coefficients for endosymbiotic gene transfer of alphaproteobacterial genes for hypothetical cell with a cell doubling time of 24 hours as a function of host cell size, host cell mitochondrial fraction and mitochondrial genome copy number per cell for a protein import cost of 2 ATP per residue. Histograms depict the selection coefficients for all genes in the endosymbiont genome. *S_R_* and *S_EGT_* have opposite signs (see methods), however to simplify the display and enable comparison the absolute value of the selection coefficients of each gene plotted.

## Fig. S4

**
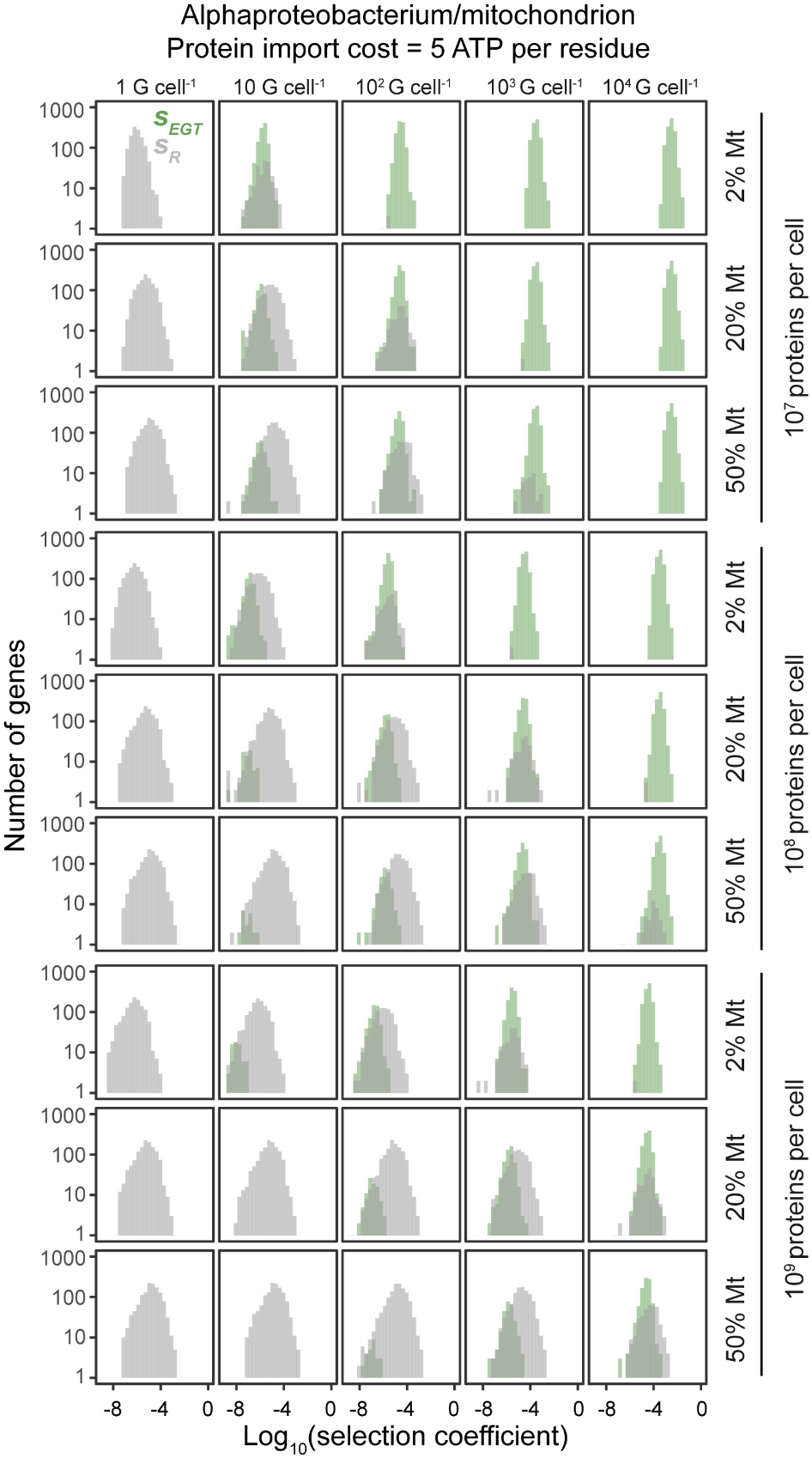
**

**Fig. S4.** The selection coefficients for endosymbiotic gene transfer of alphaproteobacterial genes for hypothetical cell with a cell doubling time of 24 hours as a function of host cell size, host cell mitochondrial fraction and mitochondrial genome copy number per cell for a protein import cost of 5 ATP per residue. Histograms depict the selection coefficients for all genes in the endosymbiont genome. *S_R_* and *S_EGT_* have opposite signs (see methods), however to simplify the display and enable comparison the absolute value of the selection coefficients of each gene plotted.

## Fig. S5

**
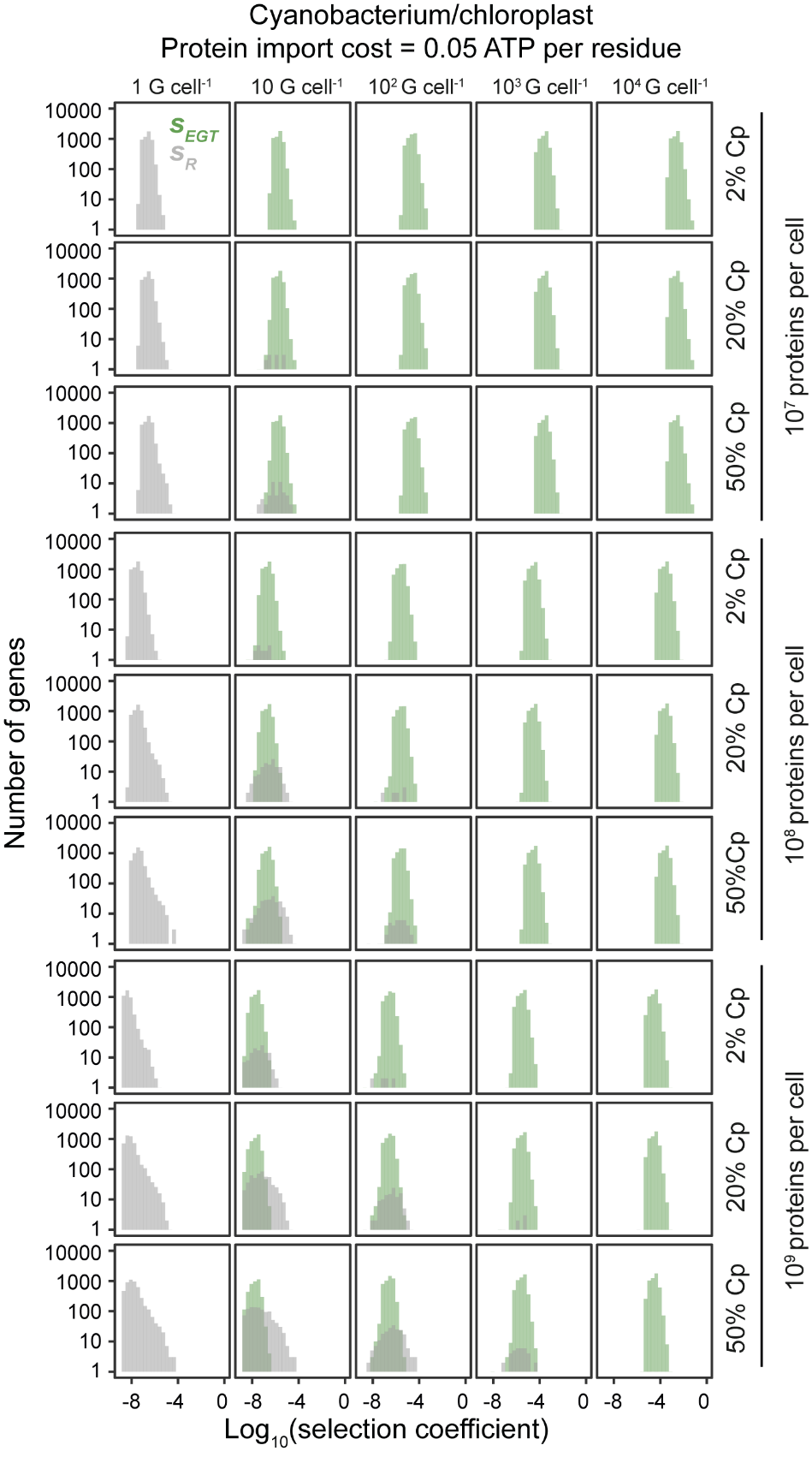
**

**Fig. S5.** The selection coefficients for endosymbiotic gene transfer of cyanobacterial genes for hypothetical cell with a cell doubling time of 24 hours as a function of host cell size, host cell chloroplast fraction and chloroplast genome copy number per cell for a protein import cost of 0.05 ATP per residue. Histograms depict the selection coefficients for all genes in the endosymbiont genome. *S_R_* and *S_EGT_* have opposite signs (see methods), however to simplify the display and enable comparison the absolute value of the selection coefficients of each gene plotted.

## Fig. S6


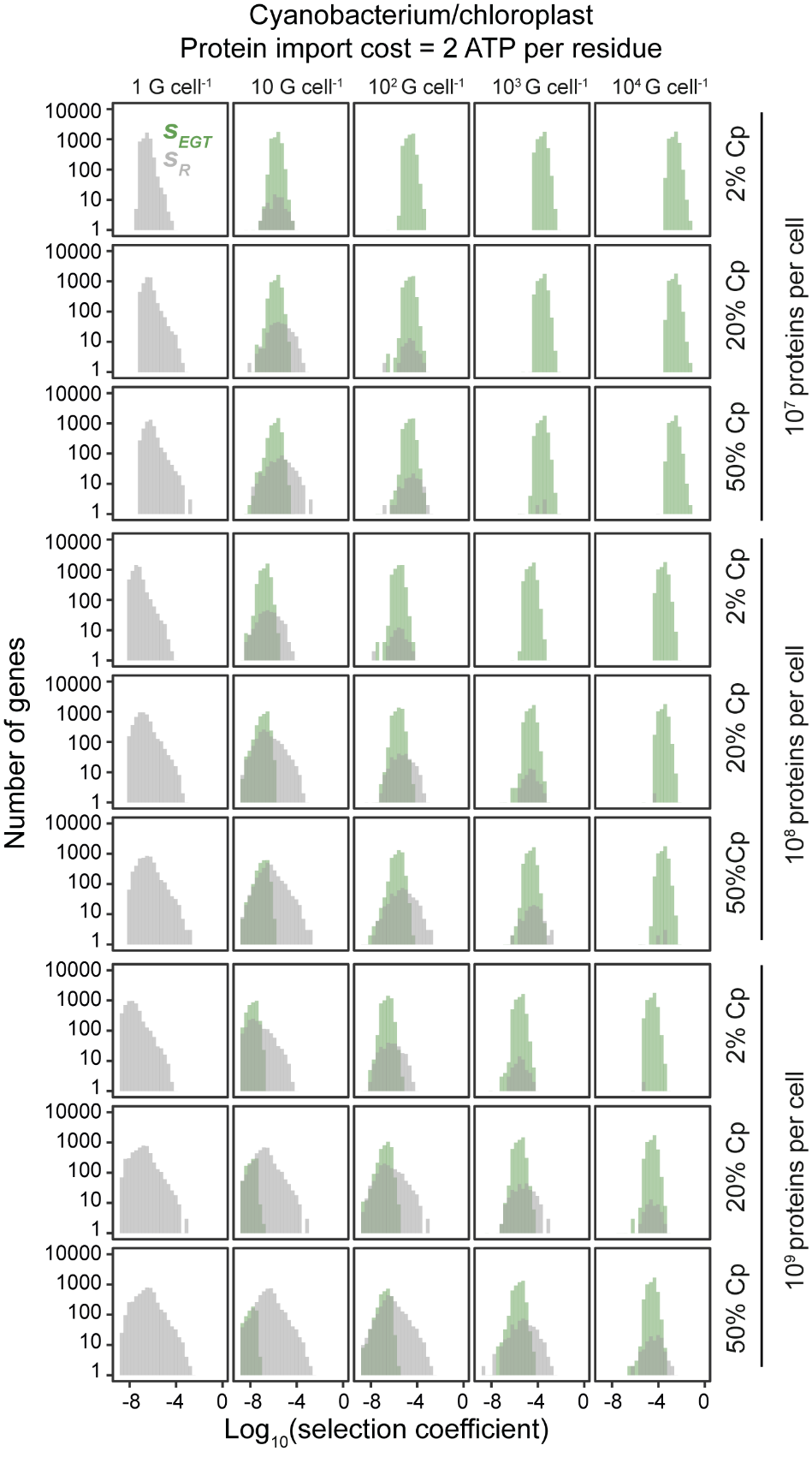


**Fig. S6.** The selection coefficients for endosymbiotic gene transfer of cyanobacterial genes for hypothetical cell with a cell doubling time of 24 hours as a function of host cell size, host cell chloroplast fraction and chloroplast genome copy number per cell for a protein import cost of 2 ATP per residue. Histograms depict the selection coefficients for all genes in the endosymbiont genome. *S_R_* and *S_EGT_* have opposite signs (see methods), however to simplify the display and enable comparison the absolute value of the selection coefficients of each gene plotted.

## Fig. S7


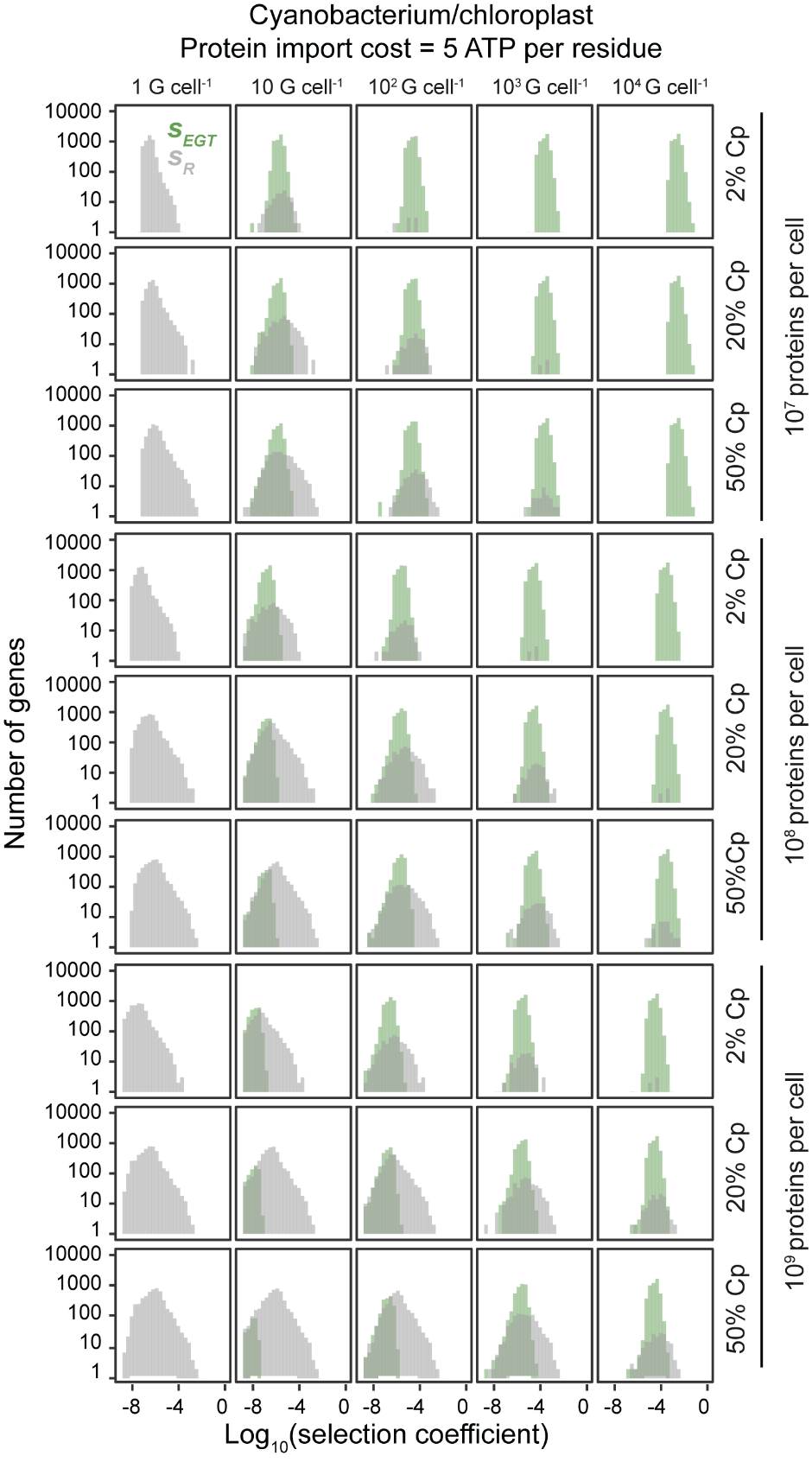


**Fig. S7.** The selection coefficients for endosymbiotic gene transfer of cyanobacterial genes for hypothetical cell with a cell doubling time of 24 hours as a function of host cell size, host cell chloroplast fraction and chloroplast genome copy number per cell for a protein import cost of 0.5 ATP per residue. Histograms depict the selection coefficients for all genes in the endosymbiont genome. *S_R_* and *S_EGT_* have opposite signs (see methods), however to simplify the display and enable comparison the absolute value of the selection coefficients of each gene plotted.

## Fig. S8


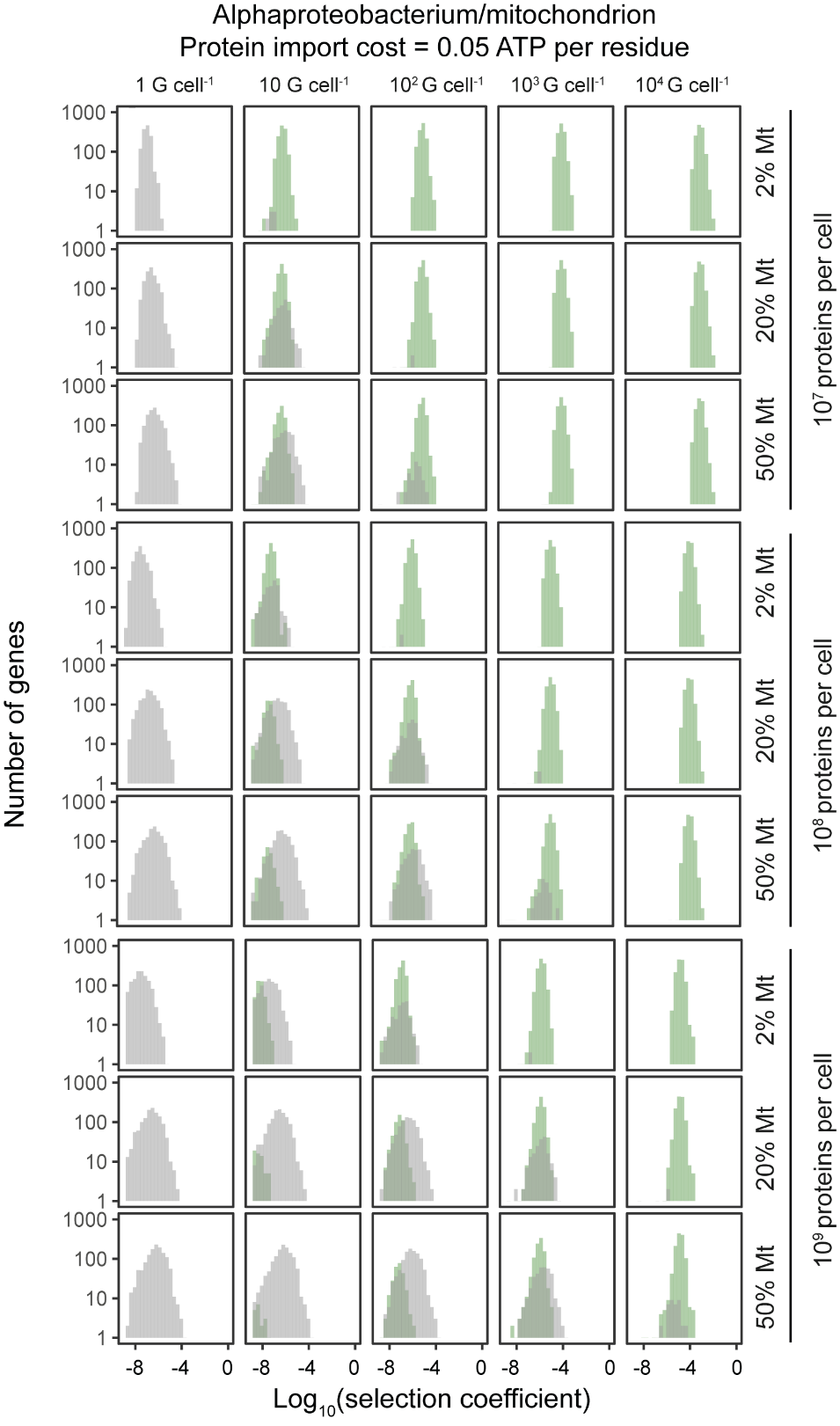


**Fig. S8.** The selection coefficients for endosymbiotic gene transfer of alphaproteobacterial genes for a hypothetical cell with a cell doubling time of 240 hours as a function of host cell size, host cell chloroplast fraction and chloroplast genome copy number per cell for a protein import cost of 0.05 ATP per residue. Histograms depict the selection coefficients for all genes in the endosymbiont genome. *S_R_* and *S_EGT_* have opposite signs (see methods), however to simplify the display and enable comparison the absolute value of the selection coefficients of each gene plotted.

## Fig. S9


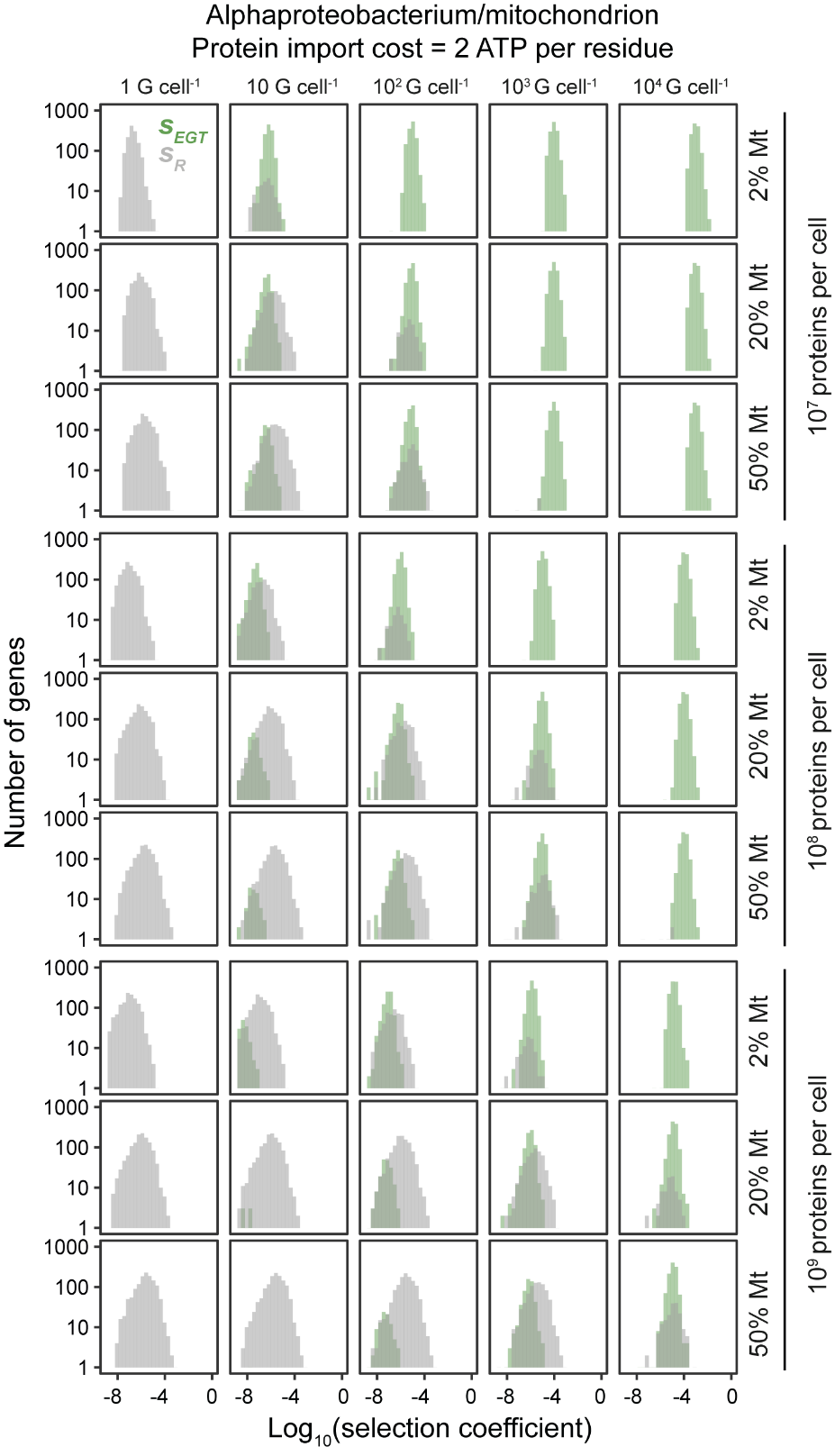


**Fig. S9.** The selection coefficients for endosymbiotic gene transfer of alphaproteobacterial genes for a hypothetical cell with a cell doubling time of 240 hours as a function of host cell size, host cell chloroplast fraction and chloroplast genome copy number per cell for a protein import cost of 2 ATP per residue. Histograms depict the selection coefficients for all genes in the endosymbiont genome. *S_R_* and *S_EGT_* have opposite signs (see methods), however to simplify the display and enable comparison the absolute value of the selection coefficients of each gene plotted.

## Fig. S10


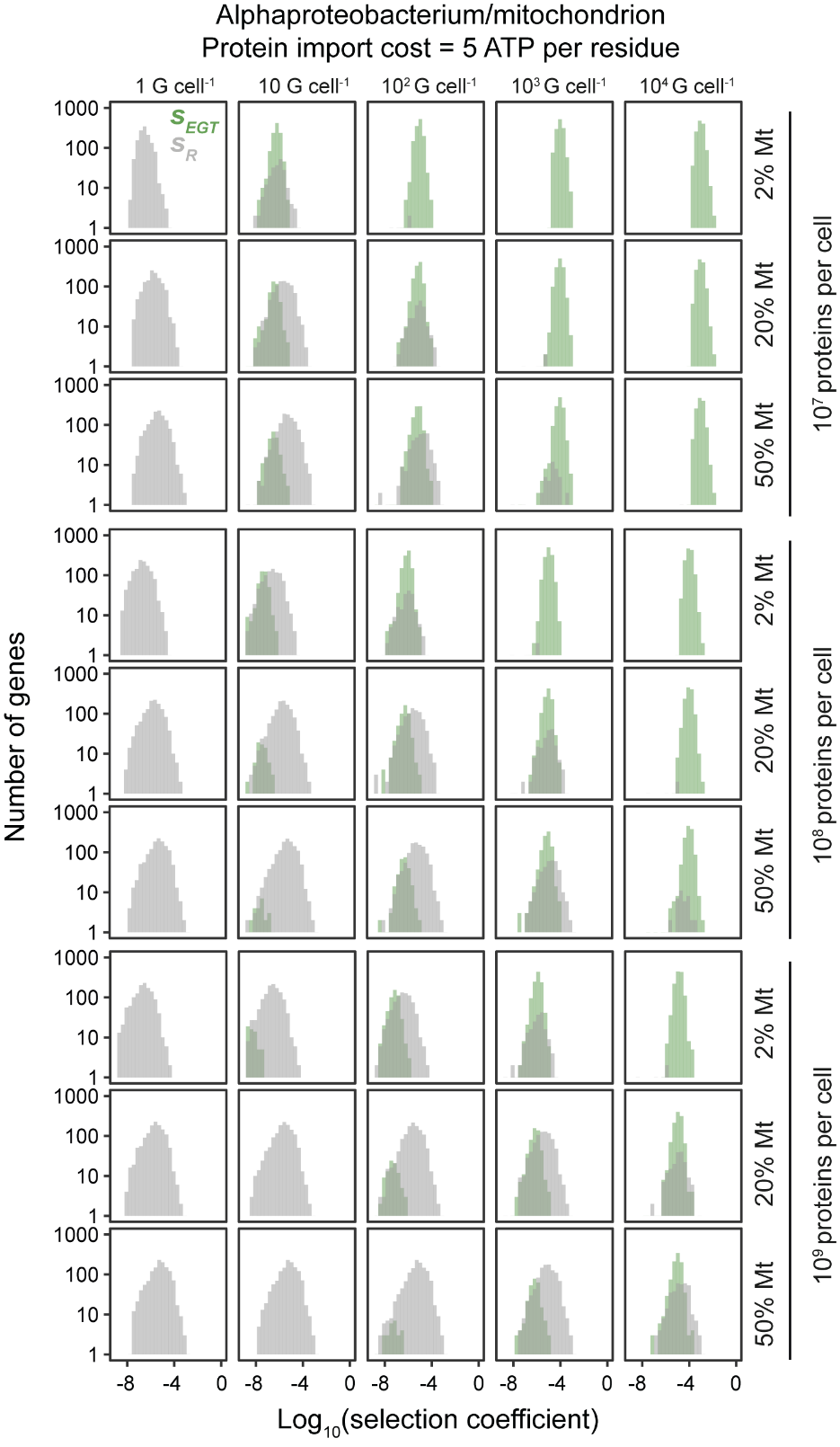


**Fig. S10.** The selection coefficients for endosymbiotic gene transfer of alphaproteobacterial genes for a hypothetical cell with a cell doubling time of 240 hours as a function of host cell size, host cell chloroplast fraction and chloroplast genome copy number per cell for a protein import cost of 5 ATP per residue. Histograms depict the selection coefficients for all genes in the endosymbiont genome. *S_R_* and *S_EGT_* have opposite signs (see methods), however to simplify the display and enable comparison the absolute value of the selection coefficients of each gene plotted.

## Fig. S11


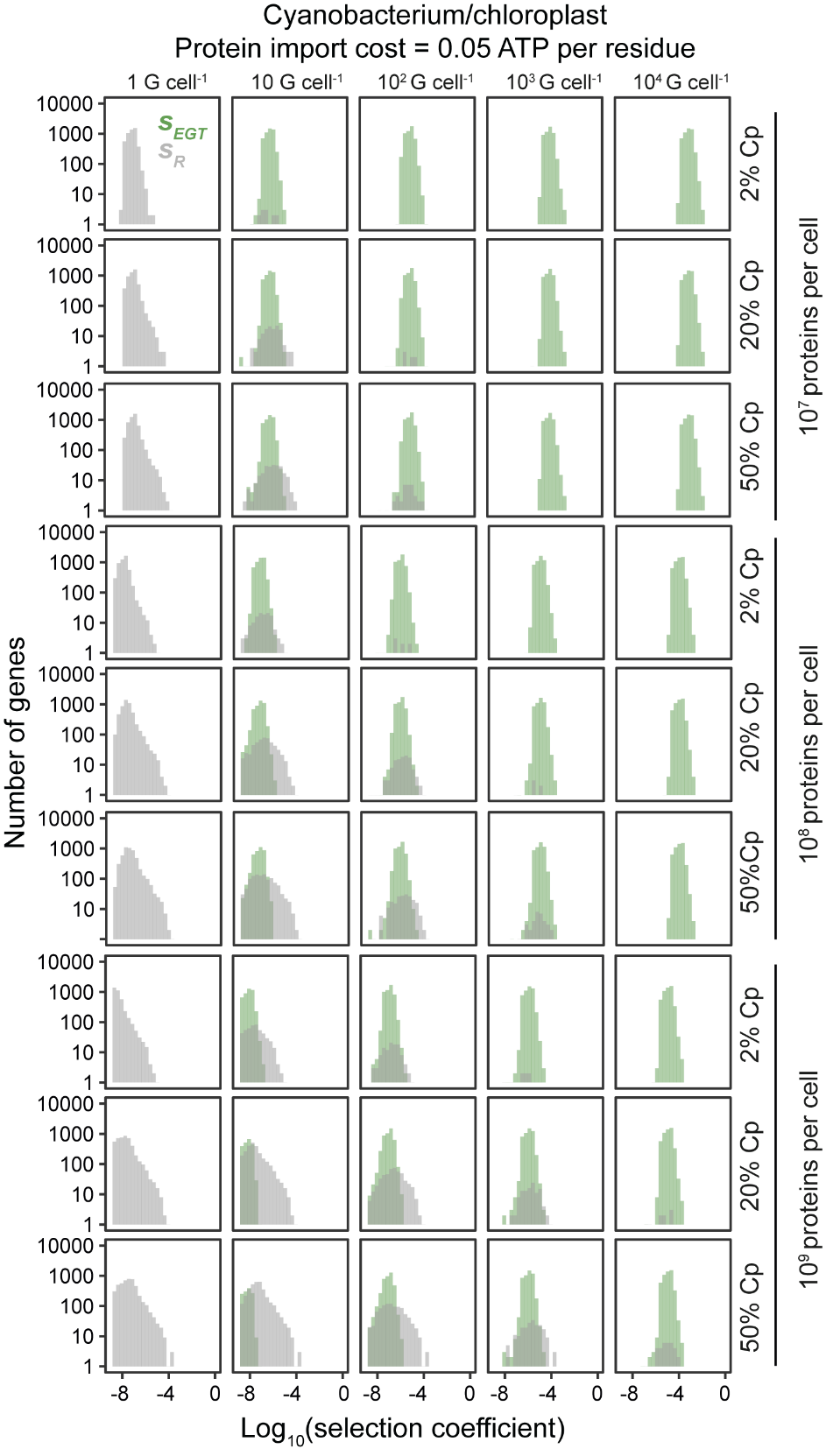


**Fig. S11.** The selection coefficients for endosymbiotic gene transfer of cyanobacterial genes for a hypothetical cell with a cell doubling time of 240 hours as a function of host cell size, host cell chloroplast fraction and chloroplast genome copy number per cell for a protein import cost of 0.05 ATP per residue. Histograms depict the selection coefficients for all genes in the endosymbiont genome. *S_R_* and *S_EGT_* have opposite signs (see methods), however to simplify the display and enable comparison the absolute value of the selection coefficients of each gene plotted.

## Fig. S12


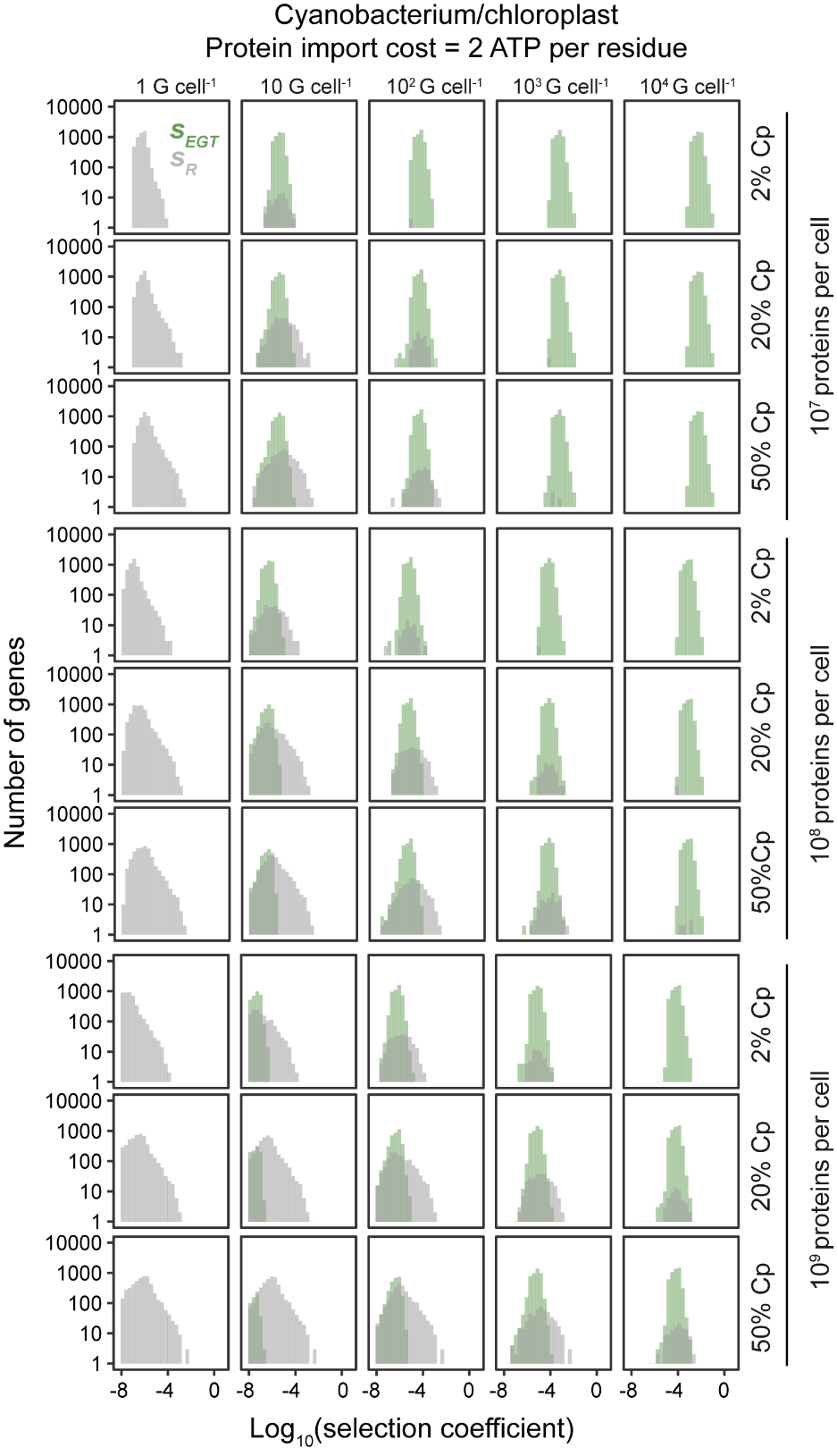


**Fig. S12.** The selection coefficients for endosymbiotic gene transfer of cyanobacterial genes for a hypothetical cell with a cell doubling time of 240 hours as a function of host cell size, host cell chloroplast fraction and chloroplast genome copy number per cell for a protein import cost of 2 ATP per residue. Histograms depict the selection coefficients for all genes in the endosymbiont genome. *S_R_* and *S_EGT_* have opposite signs (see methods), however to simplify the display and enable comparison the absolute value of the selection coefficients of each gene plotted.

## Fig. S13


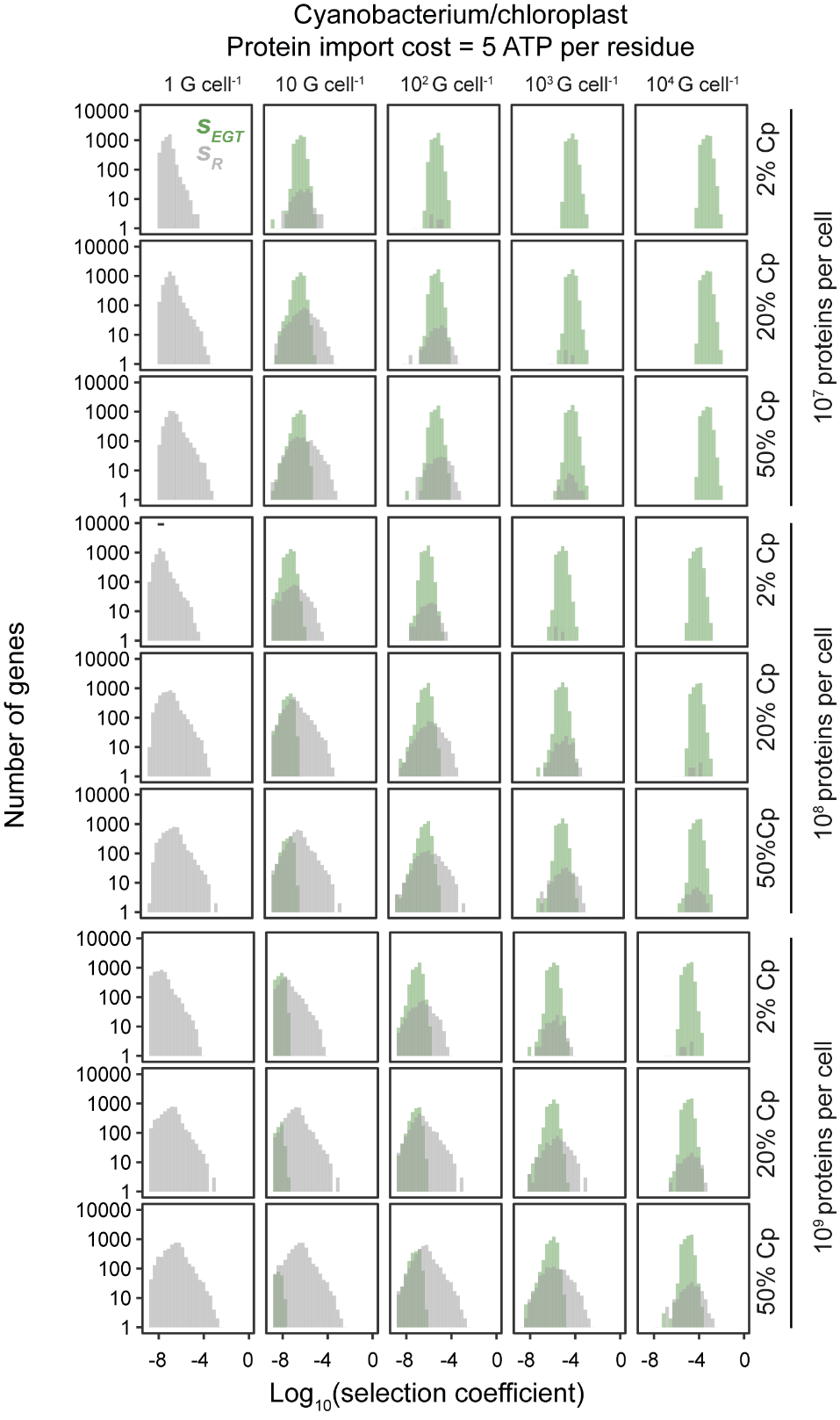


**Fig. S13.** The selection coefficients for endosymbiotic gene transfer of cyanobacterial genes for a hypothetical cell with a cell doubling time of 240 hours as a function of host cell size, host cell chloroplast fraction and chloroplast genome copy number per cell for a protein import cost of 5 ATP per residue. Histograms depict the selection coefficients for all genes in the endosymbiont genome. *S_R_* and *S_EGT_* have opposite signs (see methods), however to simplify the display and enable comparison the absolute value of the selection coefficients of each gene plotted.

## Fig. S14


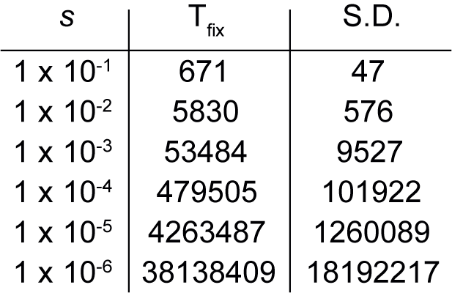


**Fig. S14** Simulated fixation times (T_fix_) and their standard deviations (S.D.) for a range of selection coefficients (s). Units are for T_fix_ are generations.

## Fig. S15


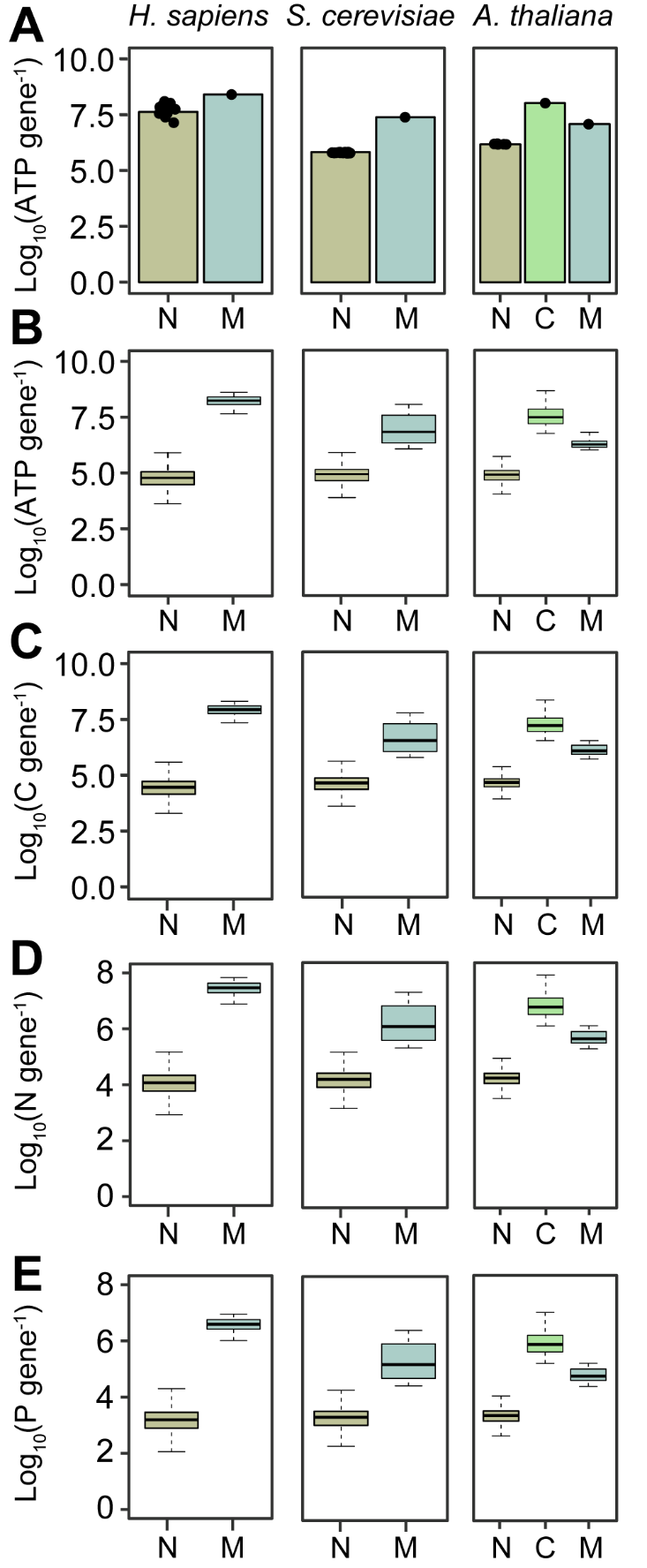


**Fig. S15.** The per-cell cost of nuclear and organellar genes in three representative eukaryotes. Part **A** and Part **B** are reproduced from the main text Figure 1 for reference. **C)** The carbon atom biosynthesis cost of nuclear (N), chloroplast (C), and mitochondrial (M) genes. **D)** The nitrogen atom biosynthesis cost of the same genes. **E)** The phosphorous atom biosynthesis cost of the same genes. Costs were computed assuming a diploid nuclear genome, a per-cell mitochondrial genome copy number of 5000, 200 and 100 for the in *H. sapiens*, *S. cerevisiae* and *A. thaliana*, respectively, and a per cell chloroplast genome copy number of 1500 in *A. thaliana*.
