# Supplementary material for: The economics of organellar gene loss and endosymbiotic gene transfer
Source: Genome Biol. 2021 Dec 20;22:345. doi: 10.1186/s13059-021-02567-w (PMC8686548; doi:10.1186/s13059-021-02567-w)
Supplement: Supplementary file 7 — Additional file 7. Review history [file 13059_2021_2567_MOESM7_ESM.docx]

Review History

**First round of review**

**Reviewer 1**

**Are you able to assess all statistics in the manuscript, including the appropriateness of statistical tests used?**

Yes, and I have assessed the statistics in my report.

**Comments to author:**

The manuscript explores ATP economics as a contributory factor to the transfer of organelle genes to the nucleus. The model suggests that, because of the high ATP cost of synthesising many organelle gene copies, it is energetically favourable to encode genes in the nucleus even when import costs and histone content are accounted for.

I previously reviewed this manuscript (positively!) for a different journal and this version is substantially stronger still; several of the questions I had at the time have been answered. This is an interesting and novel story and merits publication after some aspects have been edited.

I appreciate the consideration of protein turnover and the link with genome data. The latter, while very interesting, is however rather too bombastically phrased at the moment. The section beginning l237 repeatedly refers to "abundance" in sentences which, on their own, are open to misintepretation as "true" abundance rather than an estimate from a modern-day non-endosymbiotic relative. There are many reasons why such a relative may have different profiles of protein abundance than the pre- or proto-endosymbionts. "Abundance" should be replaced -- certainly in l237, 258, 260 and more generally if necessary -- with a less ambiguous phrasing like "estimated precursor abundance".

The title of this section "Protein abundance can explain a significant proportion of variance in loss, retention and, transfer of organellar genes" is also currently not supported by the presentation. What proportion? The result that abundance differs across gene classes is not the same as abundance predicting gene class. Either the title should be changed to e.g. "Estimated protein abundance in organelle precursors differ between lost and retained genes" or an analysis should be performed (GLM classification?) to see how much the estimated abundance genuinely predicts encoding.

This analysis revealed that the abundance of proteins encoded by genes found in extant plastid (Figure 4E) or mitochondrial (Figure 4F) genomes in eukaryotes was significantly higher than those that have been lost or transferred to the nuclear genome.

Thus, across all eukaryotes the abundance of proteins encoded by genes retained in organellar genomes is higher than the abundance of proteins encoded by genes that were either lost or transferred to the nuclear genome.

I remain concerned about the 24h doubling time assumed in the evolutionary model. I am not an expert here but I believe our closest guess at the sort of cells that were endosymbiotic "hosts" more resemble modern-day archaea, which can have extremely long cell cycles -- at least ten times longer than the current parameter*. Even if I am mistaken here, this assumed cell cycle length is an important control parameter of the evolutionary model (it sits on the denominator of the magnitude of the selection strength). This is mentioned on l542 but this is rather buried in the methods for a key parameter. It would be better to take the cell cycle time from a "best guess" ancestor (like in *) as the default parameter, compute selection coefficients for that case, and plot for a wider range.

The estimates on l481 seem very precise and surprisingly similar given the variability and magnitude of estimates for these quantities in "Physical Biology of the Cell" (Phillips et al.) and the literature on different metabolic costs of AT and GC nucleotides in organelles (e.g. https://www.frontiersin.org/articles/10.3389/fgene.2012.00175/full and refs therein) -- how much would differences in these values influence the findings of the model? Presumably linearly, but potentially several fold changes in the budget?

* https://www.nature.com/articles/s41586-019-1916-6

Iain Johnston

**Reviewer 2**

**Are you able to assess all statistics in the manuscript, including the appropriateness of statistical tests used?**

There are no statistics in the manuscript.

**Comments to author:**

This is a very interesing paper. It should be published, but the final conclusion can be modified to include Allen's insight. My discussion with the author is as follows.

line 70. The Pittis and Gabaldon paper was based on a data analysis artefact, their data is better explained by a single normal distribution rather that the 5 Gaussians the report, a classical case of overfitting. The temporal categories they distinguish (a molecular clock) do not exist. Martin WF, Röttger M, Ku C, Garg SG, Nelson-Sathi S, Landan G (2017) Late mitochondrial origin is an artefact. Genome Biol. Evol. 9:373-379.

line 100 f. Entire chloroplast and mitocondrial genomes are found in Arbidopsis and rice, very recently transferred. Huang CY, Grünheit N, Ahmadinejad N, Timmis JN, Martin W (2005) Mutational decay and age of chloroplast and mitochondrial genomes transferred recently to angiosperm nuclear chromosomes. Plant Physiol. 138:1723-1733. This underscores the glaring problem that by the measure of pure energetic economy all genes should be transferred. But some genes xware retained in organelles and always the same genes (or functions are retained -- these always center around redox balance in the bioenergetic membranes of the organelles and the genes needed to express those proteins in the organelle. Allen has been saying that since 1993, and he is right. Allen JF (1993) Control of gene expression by redox potential and the requirement for chloroplast and mitochondrial genomes. J Theor Biol 165:609-631. Allen JF (2015) Why chloroplasts and mitochondria retain their own genomes and genetic systems: colocation for redox regulation of gene expression. Proc Natl Acad Sci USA 112:10231-10238. Allen's theory nicely explains many things, includung why hydrogenosomes can relinguish their genome while still making ATP. Martin W: Gene transfers from organelles to the nucleus: Frequent and in big chunks. Proc. Natl. Acad. Sci. USA 100:8612-8614 (2003). The convergence for bioenergetic membrane functions in plastids and mitochondria would be dumbfounding otherwise, and it is not hydrophobicity…. The convergence even extends to the same ribosomal proteins. Maier U-G, Zauner S, Woehle C, Bolte K, Hempel F, Allen JF, Martin WF. Massively convergent evolution for ribosomal protein gene content in plastid and mitochondrial genomes. Genome Biol. Evol. 5:2318-2329 (2013). See also Johnston, I.G., and Williams, B.P. (2016). Cell Syst. 2: 101-111. and Allen JF, Martin WF: Why have organelles retained genomes? Cell Systems 2:70-72 (2016).

line 114. Its protein synthesis, not DNA that really costs. The DNA cost (about 3-5% of total, depending) is well within the roughly 3 fold excess of ATP that cells expend during exponential growth. Its called energy spiling or maintenance energy. It has been known for decades, since Stouthamer's early studies. Russell, J. B. (2007). The energy spilling reactions of bacteria and other organisms. J. Mol. Microbiol. Biotechnol. 13, 1-11. Russell, J. B., and Cook, G. M. (1995). Energetics of bacterial growth: balance of anabolic and catabolic reactions. Microbiol. Rev. 59, 48-62. Hoehler, T. M., and Jörgensen, B. B. (2013). Microbial life under extreme energy limitation. Nat. Rev. Microbiol. 2013, 83-94. It is this excess of energy consumption that Lynch ignores and that is the reason why his models miss the mark.

Something that has been missing from this discussion is the simple deletion bias germane to and well documented in prokaryotic genomes (Howard Ochman's papers). If the nuclear copies become even slightly expressed and minimally targeted, they can start to contribute to organellar function, then there is nothing to stop deletion bias from running its route. This is an aspect the author could mention in this paper, I think it is so far unsaid in the literature on this topic, though I might be wrong.

line 140. There is a very interesting caveat to this passage. Incredibly, it has gone almost entirely unnoticed that vitually all algae except those that made it onto land have only one plastid per cell. It has lots of genomes yes, but our common concept of many organelles per cell is founded more in tradition (and line drawings) than observation: de Vries J and Gould SB (2018) "The monoplastidic bottleneck in algae and plant evolution" Journal of Cell Science 131, jcs203414. That is not to say calculate everything over from scratch, but it does bear upon the view that selection is forcing organelle genes into the nucleus. It is more likely that transfer is just a one way street (Martin and Herrmann 1998) and that the real selection taking place is that for keeping genes in the organelle (Allen, 1993, 2015, 2016). That does not mean that there are no energetic advantages of the kind that this paper points out, but other factors might be more active in shaping the distribution we see today.

l. 178. This range is interesting. A strength.

l. 194. This is also a novel measure and a significant strength. Lynch missed this altogether.

l. 239. were that the case, all ribosomal proteins would be organelle encoded, which is not true, but it is excellent to see protein abundance taken into account, Lynch did not do that.

l. 260. This is interesting and relevant. Although, glycolysis, cytoskeleton, and the like can be very abundant. But the trend is key.

line 285 -290. This is a fantastic result and what it means is that John Allen (1993, 2015, 2016) is right. The question is why keep any genes in the organelle at all as they can all be transfereed and expressed because organell genomes are always raining down on the nucleus (Hazkani-Covo E, Martin WF: The number of independent organelle DNA insertions in genomes: connecting the dots between evolution and human health. Genome Biol. Evol. 9:1190-1203 (2017); Hazkani-Covo E, Zeller RM, Martin W: Molecular poltergeists: mitochondrial DNA copies (numts) in sequenced nuclear genomes. PLoS Genet. 6:e1000834 (2010)). In humans I thinks it 580 kb of a 16 kb mtDNA genome in the nucleus.

Thus, this paper is interesting and important. It should be published but I think the paper needs to look more carefully at what John F. Allen has said about the topic, because what this fascination paper shows in the most unequivocal terms is that John is right. The real selection pressure operating in this issue is the selection pressure that keep genes in the organelles. But the energetic approach taken here is the best that the field has to offer.

**Authors Response**

**Point-by-point responses to the reviewers’ comments:**

Reviewer 1

Comment 1

The manuscript explores ATP economics as a contributory factor to the transfer of organelle genes to the nucleus. The model suggests that, because of the high ATP cost of synthesising many organelle gene copies, it is energetically favourable to encode genes in the nucleus even when import costs and histone content are accounted for.

I previously reviewed this manuscript (positively!) for a different journal and this version is substantially stronger still; several of the questions I had at the time have been answered. This is an interesting and novel story and merits publication after some aspects have been edited.

*I would like to thank the Reviewer for their supportive comments on this and the previous version of the manuscript. Their advice has again strengthened the manuscript.*

Comment 2

I appreciate the consideration of protein turnover and the link with genome data. The latter, while very interesting, is however rather too bombastically phrased at the moment. The section beginning l237 repeatedly refers to "abundance" in sentences which, on their own, are open to misintepretation as "true" abundance rather than an estimate from a modern-day non-endosymbiotic relative. There are many reasons why such a relative may have different profiles of protein abundance than the pre- or proto-endosymbionts. "Abundance" should be replaced -- certainly in l237, 258, 260 and more generally if necessary -- with a less ambiguous phrasing like "estimated precursor abundance".

*I appreciate the reviewers concern here and have altered the text to make it clearer. I feel that the word precursor is too ambiguous and could mean metabolites (or other factors) and thus the word “protein” was retained as I am specifically talking about proteins. However, to address the reviewers concern the title of the section of the manuscript has been changed to “Proteins encoded by organellar genes have higher estimated ancestral abundance than those that have been lost or transferred to the nuclear genome”. Similarly, wherever “protein abundance” or “abundance” is discussed in this section this has now been changed to “estimated protein abundance” or “inferred ancestral abundance.”*

Comment 3

The title of this section "Protein abundance can explain a significant proportion of variance in loss, retention and, transfer of organellar genes" is also currently not supported by the presentation. What proportion? The result that abundance differs across gene classes is not the same as abundance predicting gene class. Either the title should be changed to e.g. "Estimated protein abundance in organelle precursors differ between lost and retained genes" or an analysis should be performed (GLM classification?) to see how much the estimated abundance genuinely predicts encoding. This analysis revealed that the abundance of proteins encoded by genes found in extant plastid (Figure 4E) or mitochondrial (Figure 4F) genomes in eukaryotes was significantly higher than those that have been lost or transferred to the nuclear genome. Thus, across all eukaryotes the abundance of proteins encoded by genes retained in organellar genomes is higher than the abundance of proteins encoded by genes that were either lost or transferred to the nuclear genome.

*I thank the reviewer for spotting this mistake. The title for this section has now been changed to “Proteins encoded by organellar genes have higher estimated ancestral abundance than those that have been lost or transferred to the nuclear genome.”*

Comment 4

I remain concerned about the 24h doubling time assumed in the evolutionary model. I am not an expert here but I believe our closest guess at the sort of cells that were endosymbiotic "hosts" more resemble modern-day archaea, which can have extremely long cell cycles -- at least ten times longer than the current parameter*. Even if I am mistaken here, this assumed cell cycle length is an important control parameter of the evolutionary model (it sits on the denominator of the magnitude of the selection strength). This is mentioned on l542 but this is rather buried in the methods for a key parameter. It would be better to take the cell cycle time from a "best guess" ancestor (like in *) as the default parameter, compute selection coefficients for that case, and plot for a wider range.

*I fully agree with the reviewer here. An analysis that directly addresses this point was presented in the original manuscript. i.e. the analysis of protein turn-over relative to cell division. In most organisms, protein turn-over is mediated by cell division i.e. the vast majority of proteins have half-lives that are much longer in duration than the doubling time of the cell, and thus protein turn-over occurs through replicative dilution. As protein production is the primary energy consuming activity of the cell, modelling a higher rate of protein turn-over relative to cell division time is mathematically equivalent to modelling a longer cell division time. Thus, cell division time and protein turn-over rate should be considered are two sides of the same coin. i.e. a cell with a long cell division time will have a higher rate of protein turn-over relative to cell division and a cell with a short cell division time will have a very low rate of protein turn-over relative to cell division. In this work I modelled what would happen if protein turn over exceeded cell division by a factor of 50, this is equivalent to extending a cell division time to 50 days while assuming a 24 hour protein turn-over rate. This is energetically equivalent to having a 28 minute protein turn-over rate in a 24 hour cell division cycle. This revealed that in both rapidly and slowly dividing cells (equivalent to cells with high or low rates of protein turn-over relative to their rate of cell division) it is more energetically favourable to encode the majority of organellar targeted proteins in the nuclear genome. Although, I believe the analysis in the text shows that cell division time is well accounted for in this analysis, I fully understand the reviewers request and have included the requested supplemental files that show the selection coefficient simulations for the requested 10 day cell doubling time. Consistent with the analysis of protein turn-over rate to cell division time for these slower growing cells it is still favourable to encode the majority of organellar targeted proteins in the nuclear genome under a wide range of cell sizes and organellar genome copy numbers.*

Comment 5

The estimates on l481 seem very precise and surprisingly similar given the variability and magnitude of estimates for these quantities in "Physical Biology of the Cell" (Phillips et al.) and the literature on different metabolic costs of AT and GC nucleotides in organelles (e.g. https://www.frontiersin.org/articles/10.3389/fgene.2012.00175/full and refs therein) -- how much would differences in these values influence the findings of the model? Presumably linearly, but potentially several fold changes in the budget?

*I thank the reviewer for this comment. I carefully considered this during several stages of the project. Chen et al 2016 use ~40 ATP for each nucleotide while Lynch and Marinov 2015 and Wagner 2005 use ~50 ATP for each nucleotide, I could not find any other sources for these costs or any different costs in the references provided by the reviewer. The cost value influences the model linearly such that the energetic incentive for retention or transfer would differ by 20% between the lower and higher value above. I didn’t vary this parameter in the analyses as it is always used in a product with the difference between organellar genome copy number and nuclear genome copy number (equations 8 and 9 in the manuscript) and this parameter was varied over 5 orders of magnitude from -1 to 100,000. Thus, raising the ATP cost to 50 ATP per residue would be equivalent to simulating the genome copy number difference from -1.2 to 120,000. Thus, the 20% difference in the cost of the DNA between one estimate and another imperceptibly shifts the distribution in the figures that are presented and accordingly I decided not to include it. I have included a statement concerning this in the methods section of the revised manuscript.*

Reviewer 2

Comment 1

This is a very interesing paper. It should be published, but the final conclusion can be modified to include Allen's insight. My discussion with the author is as follows.

*I thank the reviewer for their support and have modified the text of the introduction and discussion to include more discussion of John F Allen’s work.*

Comment 2

line 70. The Pittis and Gabaldon paper was based on a data analysis artefact, their data is better explained by a single normal distribution rather that the 5 Gaussians the report, a classical case of overfitting. The temporal categories they distinguish (a molecular clock) do not exist. Martin WF, Röttger M, Ku C, Garg SG, Nelson-Sathi S, Landan G (2017) Late mitochondrial origin is an artefact. Genome Biol. Evol. 9:373-379

*I thank the author for spotting this error and have removed the Pittis and Gabaldon citation.*

Comment 3

line 100 f. Entire chloroplast and mitocondrial genomes are found in Arbidopsis and rice, very recently transferred. Huang CY, Grünheit N, Ahmadinejad N, Timmis JN, Martin W (2005) Mutational decay and age of chloroplast and mitochondrial genomes transferred recently to angiosperm nuclear chromosomes. Plant Physiol. 138:1723-1733. This underscores the glaring problem that by the measure of pure energetic economy all genes should be transferred. But some genes xware retained in organelles and always the same genes (or functions are retained -- these always center around redox balance in the bioenergetic membranes of the organelles and the genes needed to express those proteins in the organelle. Allen has been saying that since 1993, and he is right. Allen JF (1993) Control of gene expression by redox potential and the requirement for chloroplast and mitochondrial genomes. J Theor Biol 165:609-631. Allen JF (2015) Why chloroplasts and mitochondria retain their own genomes and genetic systems: colocation for redox regulation of gene expression. Proc Natl Acad Sci USA 112:10231-10238. Allen's theory nicely explains many things, includung why hydrogenosomes can relinguish their genome while still making ATP. Martin W: Gene transfers from organelles to the nucleus: Frequent and in big chunks. Proc. Natl. Acad. Sci. USA 100:8612-8614 (2003). The convergence for bioenergetic membrane functions in plastids and mitochondria would be dumbfounding otherwise, and it is not hydrophobicity…. The convergence even extends to the same ribosomal proteins. Maier U-G, Zauner S, Woehle C, Bolte K, Hempel F, Allen JF, Martin WF. Massively convergent evolution for ribosomal protein gene content in plastid and mitochondrial genomes. Genome Biol. Evol. 5:2318-2329 (2013). See also Johnston, I.G., and Williams, B.P. (2016). Cell Syst. 2: 101-111. and Allen JF, Martin WF: Why have organelles retained genomes? Cell Systems 2:70-72 (2016)

*I thank the reviewer for this comment and agree with the points that are discussed. The Huang et al 2005 paper and the Martin 2003 paper have been added to the introduction at the point where the “constant stream of organellar DNA entering the nucleus” is discussed. I have also added the two Hazkani-Covo et al citations in comment 11 to this point of the manuscript. I have also added an additional paragraph to the introduction to discuss the Allen and Johnston papers in more depth and added additional discussion of the Allen papers in the discussion section as requested.*

Comment 4

line 114. Its protein synthesis, not DNA that really costs. The DNA cost (about 3-5% of total, depending) is well within the roughly 3 fold excess of ATP that cells expend during exponential growth. Its called energy spiling or maintenance energy. It has been known for decades, since Stouthamer's early studies. Russell, J. B. (2007). The energy spilling reactions of bacteria and other organisms. J. Mol. Microbiol. Biotechnol. 13, 1-11. Russell, J. B., and Cook, G. M. (1995). Energetics of bacterial growth: balance of anabolic and catabolic reactions. Microbiol. Rev. 59, 48-62. Hoehler, T. M., and Jörgensen, B. B. (2013). Microbial life under extreme energy limitation. Nat. Rev. Microbiol. 2013, 83-94. It is this excess of energy consumption that Lynch ignores and that is the reason why his models miss the mark.

*I fully agree with the reviewer and thank them for this comment.*

Comment 5

Something that has been missing from this discussion is the simple deletion bias germane to and well documented in prokaryotic genomes (Howard Ochman's papers). If the nuclear copies become even slightly expressed and minimally targeted, they can start to contribute to organellar function, then there is nothing to stop deletion bias from running its route. This is an aspect the author could mention in this paper, I think it is so far unsaid in the literature on this topic, though I might be wrong.

*I fully agree with the reviewer here. Presence of a redundant copy of a gene in the nucleus that is only slightly expressed and minimally targeted does provide an opportunity for recovery in case of gene loss in the organelle. I discussed this in the introduction to the manuscript where it states “endosymbiont genome reduction in the presence of functional compensation by lateral and/or endosymbiotic gene transfer are recurring themes in the evolution of organellar and endosymbiont genomes.” I realise this was not as clear as I intended and I have now strengthened this section to make the link pointed out by the reviewer clearer.*

Comment 6

line 140. There is a very interesting caveat to this passage. Incredibly, it has gone almost entirely unnoticed that vitually all algae except those that made it onto land have only one plastid per cell. It has lots of genomes yes, but our common concept of many organelles per cell is founded more in tradition (and line drawings) than observation: de Vries J and Gould SB (2018) "The monoplastidic bottleneck in algae and plant evolution" Journal of Cell Science 131, jcs203414. That is not to say calculate everything over from scratch, but it does bear upon the view that selection is forcing organelle genes into the nucleus. It is more likely that transfer is just a one way street (Martin and Herrmann 1998) and that the real selection taking place is that for keeping genes in the organelle (Allen, 1993, 2015, 2016). That does not mean that there are no energetic advantages of the kind that this paper points out, but other factors might be more active in shaping the distribution we see today.

*I fully agree with the reviewer here. However, even though green algae maintain a single plastid they maintain very high copy numbers of the organellar genome in that plastid often distributed in several nucleoids in the single plastid. Thus, they are well described by the models in this work, as essentially the energetic saving (or not) of transferring a gene to the nuclear genome boils down to the ratio between the organellar genome copy number and the nuclear genome copy number. Where it has been measured unicellular green algae have ~100 chloroplast genome copy numbers per cell in normal lab growth conditions (e.g. Gallaher et al 2017 The Plant Journal 93: 545-565).*

Comment 7

l. 178. This range is interesting. A strength.

*Thank you.*

Comment 8

l. 194. This is also a novel measure and a significant strength. Lynch missed this altogether.

*Thank you.*

Comment 9

l. 239. were that the case, all ribosomal proteins would be organelle encoded, which is not true, but it is excellent to see protein abundance taken into account, Lynch did not do that.

*Thank you.*

Comment 10

l. 260. This is interesting and relevant. Although, glycolysis, cytoskeleton, and the like can be very abundant. But the trend is key.

*Thank you.*

Comment 11

line 285 -290. This is a fantastic result and what it means is that John Allen (1993, 2015, 2016) is right. The question is why keep any genes in the organelle at all as they can all be transfereed and expressed because organell genomes are always raining down on the nucleus (Hazkani-Covo E, Martin WF: The number of independent organelle DNA insertions in genomes: connecting the dots between evolution and human health. Genome Biol. Evol. 9:1190-1203 (2017); Hazkani-Covo E, Zeller RM, Martin W: Molecular poltergeists: mitochondrial DNA copies (numts) in sequenced nuclear genomes. PLoS Genet. 6:e1000834 (2010)). In humans I thinks it 580 kb of a 16 kb mtDNA genome in the nucleus.

*Thank you! These additional citations have been added to the relevant section of the introduction.*

Comment 11

Thus, this paper is interesting and important. It should be published but I think the paper needs to look more carefully at what John F. Allen has said about the topic, because what this fascination paper shows in the most unequivocal terms is that John is right. The real selection pressure operating in this issue is the selection pressure that keep genes in the organelles. But the energetic approach taken here is the best that the field has to offer.

*I thank the reviewer again for their supportive comments. I have added additional discussion of John Allen’s work to the revised introduction and discussion sections of the manuscript.*

**Second round of review**

**Reviewer 1**

The author has, to my eyes, done a great job responding to my comments and the other reviewer's. I had one additional thought which I hope will not come as a surprise to him. Please note that this came up as a result of my delayed thinking about the original manuscript and not as a result of the revisions.

All mechanisms of gene transfer I know involve a transient period where the gene is encoded both in the organelle and the nucleus (e.g. lyse one organelle of many, incorporate sequence into nucleus, then slowly atrophy from other organelles over generations). If DNA cost of encoding is important for fitness, isn't there a big fitness hurdle in this transient, which has to pay twice over? Bigger than either individual encoding strategy? Is it reasonable that organisms could jump that hurdle to eventually get to the lower cost version?

NB I'm talking purely about the encoding costs here, not the import costs. Say it costs me $10 to encode in oDNA, $1 to encode in the nucleus, and $5 to import. Gene transfer takes me down the following route:

oDNA encoding and organelle expression = $10

oDNA encoding and organelle expression plus nuclear encoding (possibly without nuclear expression) = $11

Nuclear encoding and expression = $6

My question is, how does evolution overcome that bump from (oDNA encoding + organelle expression) to (oDNA encoding + organelle expression + nuclear encoding)?

If this can be addressed (ideally quantitatively given the established evolutionary model) I am very happy to recommend this work for publication.
